# Domain Adaptation-enhanced searchlight: enabling classification of brain states from visual perception to mental imagery

**DOI:** 10.1186/s40708-025-00263-0

**Published:** 2025-06-28

**Authors:** Alexander Olza, David Soto, Roberto Santana

**Affiliations:** 1 Intelligent Systems Group, University of the Basque Country (UPV/EHU), Donostia-San Sebastián, Spain; 2 Consciousness Group, Basque Center for Cognition, Brain and Language (BCBL), Donostia-San Sebastián, Spain; 3 Ikerbasque, Basque Foundation for Science, Bilbao, Spain

**Keywords:** Domain Adaptation, Brain decoding, FMRI, Searchlight

## Abstract

In cognitive neuroscience and brain-computer interface research, accurately predicting imagined stimuli is crucial. This study investigates the effectiveness of Domain Adaptation (DA) in enhancing imagery prediction using primarily visual data from fMRI scans of 18 subjects. Initially, we train a baseline model on visual stimuli to predict imagined stimuli, utilizing data from 14 brain regions. We then develop several models to improve imagery prediction, comparing different DA methods. Our results demonstrate that DA significantly enhances imagery prediction in binary classification on our dataset, as well as in multiclass classification on a publicly available dataset. We then conduct a DA-enhanced searchlight analysis, followed by permutation-based statistical tests to identify brain regions where imagery decoding is consistently above chance across subjects. Our DA-enhanced searchlight predicts imagery contents in a highly distributed set of brain regions, including the visual cortex and the frontoparietal cortex, thereby outperforming standard cross-domain classification methods. The complete code and data for this paper have been made openly available for the use of the scientific community.

**Supplementary Information:**

The online version contains supplementary material available at 10.1186/s40708-025-00263-0.

## Introduction

The mapping of brain activation patterns to their corresponding cognitive states is known as brain decoding. Methods for brain decoding are instrumental in understanding the brain processes involved in cognition [1] and therefore an increasing number of works address the question of solving brain decoding tasks with machine learning algorithms [2–4]. While “brain decoding” can encompass various tasks, including visual stimulus reconstruction, this study specifically focuses on the classification of neural patterns associated with visual perception and mental imagery.

Specifically, the degree to which visual imagery depends on the same neural mechanisms as visual perception remains a subject of ongoing research. Previous studies using standard multivoxel pattern classification analyses (MVPA) showed that a classifier trained to discriminate the contents of perception in the visual cortex can predict the contents of visual imagery [5–8]. However, evidence that perception and imagery share neural representation in frontoparietal areas is scarce [9, 10].

In the present study, we hypothesize that failures to reveal similar neural representations may be due to the classification pipeline not accounting for the different distributions of the data across perception and imagery domains. Several types of distribution shift can occur between perception and imagery. A covariate shift is likely, in which voxel-wise activation patterns differ in magnitude or structure across conditions despite perceiving/imagining the same concept. Additionally, a concept shift could arise, where the neural representation of a category changes between perception and imagery due to differences in sensory input versus internal generation. Consequently, we claim that Domain Adaptation (DA) methods [11, 12] are an adequate approach to alleviate hypothesized distribution shifts and enhance the transferability of classifiers trained in visual perception to imagery testing data. Section 2 includes a formal definition of covariate and concept shift.

Thus, we investigate the impact of different DA approaches in a particular fMRI cross-domain experiment where subjects were asked to solve perceptual and imagery tasks. Cross-domain classification tasks involving imagery are the basis for Brain-Computer Interfaces (BCIs) [13] and have been addressed before [6, 7, 14, 15] but, to our knowledge, the literature is scarce when it comes to applying DA techniques to such problems using fMRI data. Furthermore, rather than restricting our analysis to the visual cortex, we explore the whole brain using one local neighbourhood classifier per voxel (a searchlight procedure, as introduced by [16]). In this procedure, the brain is partitioned into thousands of overlapping spheres and the voxels of each sphere are assigned to a classifier. The searchlight method combats the curse of dimensionality by limiting the radius of the sphere, and offers an anatomically interpretable output.

In this paper, we integrate a DA technique into the searchlight method to unveil common neural representations for perception and imagery throughout the brain.

In summary, the contributions made in this paper are as follows.

1. First extensive evaluation and comparison of well-established DA techniques on cross-domain classification problems defined on fMRI data, considering several brain Regions Of Interest (ROIs) and two independent datasets.
2. Introducing the DA-enhanced searchlight approach using two different DA techniques on the whole brain, to gain more understanding on the nature and location of the domain-shift.

## Methodological background

We define a domain *D* as a combination of input and output spaces and an associated probability distribution: $(X,Y,p_D)$. In the simplest case, there is a source domain *S* with enough data to build a reliable learner, and our aim is to generalize to a different target domain *T* for which there is limited knowledge. In this paper, we consider the situation in which both domains share input and output spaces, meaning that they have the same dimensionality and live in the same space, but data come from different probability distributions $p_S$ and $p_T$.

From the ML point of view, the problem we are interested in is risk minimization in the target domain, given a set of data $(x_i,y_i)\sim p_S(x,y)$ from the source distribution (following notation from [17]). Although the formulation is valid for a general output space *Y*, we will restrict ourselves to binary classification. In this setting, we would like to find the best learner $h^\star$, defined as the one with minimum expected loss *l* under the target distribution, $h^\star = arg\underset{h\in \mathcal {H}}{min} R_T(h)$, where *h* is any learner included in a given hypothesis space $\mathcal {H}$. The expression for the risk includes a term $p_T(x_i,y_i)$, that represents the probability of the source samples under the target distribution (Eq. 1, left-hand side).

Introducing into the integral the probability of the observations coming from their actual source distribution $p_S$, we arrive to an expectation under $p_S$ of the loss multiplied by the ratio of target to source distributions (Eq. 1 right-hand side).

1 $$\begin{aligned} R_T(h)=\sum _{y\in Y}\int _X l(h(x)|y)p_T(x,y)dx = \sum _{y\in Y}\int _X l(h(x)|y)\frac{p_T(x,y)}{p_S(x,y)}p_S(x,y)dx \end{aligned}$$

In practice, this expectation can be estimated using the sample average (empirical risk minimization) [18]. Depending on the structure of $p_S$ and $p_T$, the quantity $p_T(x,y)/p_S(x,y)$ can be further simplified by factoring into conditional and marginal distributions, giving rise to special cases of distribution shifts. For example, if the prior probabilities of *y* differ between domains, but the conditionals *p*(*x*|*y*) are close enough, we have a case of prior shift. Other particular settings include covariate shift (similar posterior distributions *p*(*y*|*x*) for *T* and *S*) and concept shift (similar data distributions *p*(*x*) but $p_T(y|x)\ne p_S(y|x)$). In general, though, the data-set shifts have a more complicated and unknown structure. Depending on the nature of the distribution shift, certain DA approaches may be more suitable than others [19].

DA requires some previous knowledge of the target domain, be it in the form of some labelled (supervised DA, also known as few-shot learning) or unlabelled (unsupervised DA) instances from the target distribution. The amount of target domain knowledge incorporated into a DA technique is usually too scarce to build a model that relies on that information alone. In fact, for most DA applications, acquiring (or labelling) data from the target domain is difficult and/or expensive, but there is enough source domain data to build and validate a model with an adequate performance of unseen samples from that same source domain.

Given that such a model is achieved, generalization to novel examples from the target distribution can be tackled by several strategies, namely (a) instance-based, (b) feature-based and (c) parameter-based approaches, as summarized in Table 1. The naïve inclusion of the available target domain knowledge as additional training samples can also be considered as a rudimentary form of DA.

**Table 1:** Summary of main approaches to Domain Adaptation

Main approaches to Domain Adaptation		
Methods and key ideas	Examples	
	Supervised	Unsupervised
1. Instance-based $\bullet$ Measure the difference between each source domain and target domain instances. $\bullet$ Adjust the target domain instance contributions during training.	$\bullet$ Balanced Weighting [20] $\bullet$ TrAdaBoost [21] $\bullet$ IWN [22]	$\bullet$ KMM [23] $\bullet$ ULSIF [24] $\bullet$ RULSIF [25]
2. Feature-based $\bullet$ Look for a space of common features with respect to the task on source and target domain. $\bullet$ Build a representation under which $p_S$ and $p_T$ are similar.	$\bullet$ FA and PRED [26] $\bullet$ Fine-tuning [27]	$\bullet$ SA [28] $\bullet$ MCD [29] $\bullet$ DANN [30] $\bullet$ Deep CORAL [31]
3. Parameter-based $\bullet$ Adapt the parameters of a pre-trained source-only model to build a suitable model with the same structure on the target domain.	$\bullet$ Regular Transfer [32]	

## Related work

In this section, we review a number of works related to our research. We cover: (1) ML techniques for investigating commonalities in brain mechanisms for visual and imagery tasks; (2) DA methods applied to other neuroscience scenarios.

### ML techniques for investigating commonalities in brain mechanisms for visual and imagery tasks

Numerous works have aimed to elucidate whether visual perception and imagery arise from similar brain mechanisms. Since the development of brain imaging, early studies using multivariate pattern analysis (MVPA) attempted direct transfer from perception to imagery [6, 7, 14, 33], but consistently reported low generalization, pointing to distribution shifts between tasks. More recent work has explored voxel-wise encoding models [8], deep learning-based decoders [34], and representational similarity analyses [35], often reporting performance gaps or task-specific divergences [36]. These findings suggest that while high-level representations may overlap, their expression differs across domains.

Despite these insights, no prior work has systematically applied or compared DA techniques to address these distributional differences. Our study fills this gap by benchmarking standard DA methods to align perception and imagery data, using both ROI and searchlight analyses to localize effects and assess individual variability.

### Other applications of DA in neuroscience

DA has been applied in cross-device diagnosis [37, 38] and cross-subject EEG decoding [39, 40], often using feature or instance-based strategies [41, 42]. In [43], the authors compare the performance of four DA techniques in a cross-device MRI image segmentation task using support vector machines.

While deep learning DA is increasingly common for tasks like brain lesion segmentation [44], DA applications to fMRI remain underdeveloped.

A recent survey [45] advocates for including DA in the computational neuroscientist’s toolkit, highlighting the need to test state-of-the-art DA methods in scenarios of complex data such as neuroimaging.

To this day, few studies have tackled domain shift across cognitive conditions (e.g., perception vs. imagery), and even fewer have explored whether existing DA tools can enhance generalization in this context. Our work addresses this by conducting a thorough comparison of DA methods for a cross-condition fMRI classification problem, aiming to both quantify and reduce the domain shift.

## Description of the cross-domain analysis

**Fig. 1:**
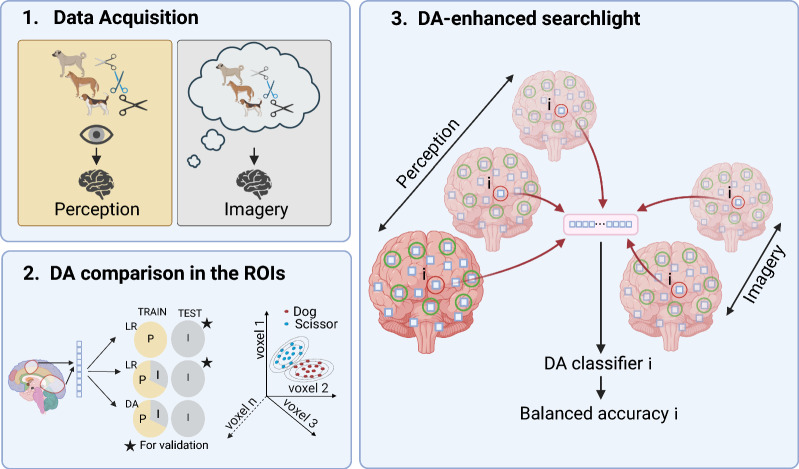
Diagram of the experimental pipeline. Data acquisition: In the perception phase, fMRI data from 18 healthy subjects was acquired while they were presented with pictures of Living (Dog) and Non-living (Scissor) items. In the imagery phase, fMRI scans were recorded while the participants vividly imagined items from each category. DA comparison in the ROIs: The voxels from 14 anatomical Regions of Interest (ROIs) were used to compare 15 DA methods against a Logistic Regression (LR) baseline trained solely on perception and an alternative LR trained on perception and imagery. All the methods were tested on imagery. The comparison was repeated on a publicly available multiclass dataset. DA-enhanced searchlight: A local DA classifier was trained on the vicinity of each voxel in the brain, using data from perception and imagery, to produce a three-dimensional map of classifier accuracy. Created in BioRender. O, A. (2025) https://BioRender.com/u30c785

Figure 1 summarizes the experimental pipeline detailed in the following subsections.

### Experimental setup

We quantified the contribution of 15 different DA algorithms to a binary cognitive state classification task using 18 data-sets, each containing around 9000–14000 anatomically selected voxels from approximately 500–600 fMRI images of a specific subject gathered in independent realizations of the same experiment, described in detail in [46]. The voxels come from 14 ROIs, the exact dimensionality of which is subject-dependent (Supplementary Table A2). The ROIs were extracted using FreeSurfer [47], and their names are listed in Supplementary Table A1.

The experiment from which the data-sets were collected consisted of two phases: (1) perception and (2) imagery. In the perception step, each participant was presented with living (Dog) and non-living (Scissor) images from the THINGS [48] database. In the imagery stage, they received auditory cues instructing them to vividly imagine an object from one of those two categories for several seconds. The goal was to classify imagery instances into living or non-living, leveraging a classifier trained mostly on perception instances.

In the perception phase, subjects participated in 4 runs of scanning. Each run was composed of 14 trials, 7 for each category (living/non-living). In each trial, participants observed a sequence of 12 images belonging to the same category. There was an 8.5-second rest period between trials, to ensure separation between brain activity patterns associated with each category. The imagery phase followed an analogous structure but consisted of only 2 runs. Although it is sometimes customary to average volumes within each trial to exploit the intermixing caused by hemodynamic delays, our study avoids this aggregation to maintain a sufficient number of samples for training and testing the classifiers.

Data preprocessing is thoroughly described in [46]. The first experimental volume, obtained after the heat-up phase, served as a functional reference. The rest of the volumes of each participant were co-registered to their corresponding reference volume. To minimize drift, the fMRI time-series was linearly detrended for each run. To capture peak neural activity in response to the presented stimuli, fMRI volumes from 5 to 18 s following the stimuli onset were retained (7 volumes per trial). The chosen samples were then independently stacked for region of interest (ROI) delineation. Next, the stacked perceptual volumes were z-scored by subtracting each voxel’s mean value and dividing by its standard deviation. Standardization of the imagery run scans was performed using the mean and standard deviation of the perceptual voxels to maintain a consistent and comparable scale across both conditions, facilitating analysis of the generalizability of information from perception to mental imagery. This preprocessing decision was maintained in our work in order to remain comparable with [46]. The same standardization was used for all DA methods under study and, crucially, for the baseline classifiers as well. Thus, all methods compared in this study are equally affected by this preprocessing procedure, isolating the effect of DA techniques. In [46], the dataset was acquired with real-time neurofeedback applications in mind, and HRF convolution was avoided to prevent delays in delivering feedback signals. We kept their preprocessing procedure intact to preserve comparability.

We also performed the same analysis in a multiclass setting using the publicly available dataset “Generic Object Decoding” [34], that contains fMRI scans from 5 subjects in both the visual perception and imagery domains. In this dataset, the classifiers decode stimulus identity instead of Living/Non-Living status. The dataset description and the obtained results are shown in Appendix B.2.

### Machine learning task and validation framework

Taking the delay of the hemodynamic response and the timing of stimulus presentation into account, there was not a one-to-one correspondence between stimuli and fMRI images within each trial, but those from different trials were safely considered independent. Thus, in all our analysis, we avoided data leakage ensuring that all instances of the same trial belonged either to the train or the test set.

We used 100 different partitions of the data to validate our methodology. Each partition determined the exact assignment of instances into train or test sets. The source domain (perception) train/test ratio was 4:1, with approximate class stratification and observing the trial constraint. The number of trials per train/test partition is shown in Supplementary Table A3. As for the target domain, we randomly selected $N_t$ instances with approximate class stratification to compose the training set, discarded the rest of the instances belonging to those trials, and used the remainder for testing. $N_t$ is a parameter of the methodology, which we varied from 10 to 100 in steps of 10. This subsampling simulates scenarios where target samples are scarce or expensive to collect. Although the datasets under study contain sufficient amounts of target data, this consideration is applicable to many neuroimaging contexts, particularly when aiming for applications like real-time neurofeedback. Our setup was designed to foster the applicability of our methodology to those scenarios.

For each train/test split, we fitted a baseline estimator *h* on the source training set, as well as another instance of the same standard classifier on the union of source and target training sets ($h_{NAIVE}$). We evaluated their performance on the target test set. *h* is considered to be the experiment baseline, and $h_{NAIVE}$ accounts for the effect of naïvely incorporating limited target data into a standard classifier without proper domain adaptation techniques. To assess the contribution of a specific DA technique, we fitted a new instance of the chosen estimator on the source domain training set $(X_s, y_s)$ alone and incorporated the target training instances $(X_t, y_t)$ through said DA technique, obtaining a new estimator, $h_{DA}$, which we evaluated on the test sets of the target domain. We chose Logistic Regression as an estimator due to its simplicity, computational speed and compatibility with all DA methods under study.

For each subject, we compared the performance of 15 DA approaches on the union of all ROIs using the implementations in the ADAPT [49] library. Appendix A.2 contains a brief description of each method. Due to the size of the dataset (with around 10 000 features), the Deep Learning methods - Fine-tuning (FT), Domain-Adversarial Neural Networks (DANN), Maximum Classifier Discrepancy (MCD) and Deep Correlation Alignment (DCORAL) - benefit from a prior dimensionality reduction step. Thus, we performed Independent Component Analysis (ICA) retaining 100 components. We also tested alternative dimensionality reduction methods retaining 100 components, specifically Truncated Singular Value Decomposition (TSVD) and Sparse Random Projection (SRP), as well as using the raw features. For dimensionality reduction, we used the implementations in scikit-learn 1.5.2 with default parameters.

Excluding the Deep Learning methods, we also compared the DA approaches in the Fusiform Gyrus (FFG), a high-level visual area known to encode animacy and category-specific information, and the Medial Orbital Gyrus (MOG), a control region which is not typically associated with visual perception or imagery..

As mentioned above, in the first place, we evaluated the contribution of each DA technique to our task in the different subjects using the voxels from all 14 ROIs. We also compared DA techniques in a task-relevant ROI (the FFG) and in a control region (the MOG). Then, based on the comparison results, we also evaluated the performance of the best technique beyond the 14 ROIs, considering all the voxels from the whole brain. This whole-brain analysis was conducted using the searchlight [16] procedure. To evaluate the robustness of the neuroscientific implications of our findings, we repeated this procedure using an alternative DA method.

### Evaluation metrics and statistical validation

In this section, we detail the validation strategies, both for the initial comparison of DA techniques at the ROI level and for the DA-enhanced searchlight.

#### Initial comparison of DA techniques

The evaluation criteria on which our comparisons are based are detailed hereafter. First, note that the methodology was constructed in such a way that the training of *h*, $h_{NAIVE}$ and $\{h_{DA}\}_{DA=1}^{15}$ were performed on exactly the same data, and the same is true for the evaluation phase of all estimators. During the 100 repetitions of the process, the balanced accuracies of each of the 17 classifiers involved in the comparison were measured on the test set of the target domain, obtaining the 17 corresponding performance vectors of size 100. Those 17 balanced accuracy vectors of *h*, $h_{NAIVE}$ and $\{h_{DA}\}_{DA=1}^{15}$ can be thought of as paired observations.

To quantify the amount of information needed to actually profit from DA methods, we applied our methodology with a varying amount of target domain instances $N_t$ between 10 and 100 in steps of 10, conducting the same comparison. By doing that, we determined the effect of incorporating a growing amount of target training instances into both $h_{NAIVE}$ and $h_{DA}$; that is, whether $h_{DA}$ profits from this information more than $h_{NAIVE}$ does.

Hence, our results are in the form of 10 balanced accuracy tables per subject, one corresponding to each value of $N_t$. Each table has 17 columns (one per ML approach) and 100 rows (one per data partition).

Firstly, we concatenated the 10 tables pertaining to each subject and conducted an all versus all statistical comparison based on the Friedman aligned ranks test, with a Shaffer correction for multiple comparisons. The non-parametric character of the Friedman test avoids distributional assumptions on our results, and the Shaffer correction controls the family-wise error rate with a low computational cost compared to other correction methods.

We also concatenated all the results (i.e. each of the 180 tables pertaining to each subject and value of $N_t$) to obtain a global comparison between all methods.

All the statistical analyses detailed in this section were based on the implementations in the R library scmamp [50].

#### Evaluation of the searchlight analysis

For the searchlight analysis, we used the same data partitioning methodology as for the ROI-based analysis. In this case, each sample contains a whole-brain three-dimensional image with around 40,000 voxels (the exact number of which is subject-dependent—see Supplementary Table A2). Each voxel is a cube with a 3 mm side, and we conducted the procedure using spheres with radius 9 mm, 12 mm and 15 mm to inspect the robustness of our results. In terms of voxels, the final size of the spheres depends on their location, with spheres in deeper brain locations containing a maximal number of voxels and spheres on the surface having the minimum size. With a radius of 12 mm, the mean and standard deviation of the number of voxels per sphere is $222\pm 47$. Spheres of 9 mm and 15 mm contained $109\pm 21$ voxels and $427\pm 99$ voxels respectively. We used algorithms RTLC and BW (see C.2 for the results of the latter).

We fitted three independent Logistic Regression classifiers on each sphere:

1. *h*: Trained only on the samples from the source domain to establish a baseline.
2. $h_{NAIVE}$: Trained on the union of samples from the source domain and 100 samples from the target domain, to assess the effect of including imagery data without DA.
3. $h_{DA}$: Incorporating the same 100 samples from the target domain into *h* via the RTLC or BW algorithm.

All classifiers were tested on the same holdout instances from the target domain. *h* was also tested on a hold-out set from the source domain. The procedure was repeated for 100 different data partitions, and the mean balanced accuracy per sphere was computed, obtaining a three-dimensional brain map of performance scores for each subject and classifier. To carry out these analyses, we integrated some functionality from ADAPT into the searchlight tools from the Nilearn library [51].

We also performed a control searchlight procedure where the target labels were altered using random permutations, hence computing the voxel-wise empirical null distribution.

To be able to extract meaningful anatomical insights and make comparisons between subjects, each score map was transformed to the standard Montreal Neurological Institute (MNI) space using the software FSL [52]. Subsequently, the maps from all the subjects were merged into a single four-dimensional image.

The statistical validation of our results employed the randomise technique [53] as implemented in FSL. This permutation-based strategy requires only minimal assumptions for validity (in our use case, exchangeability of the subjects and a symmetric distribution of the centred maps at a voxel level) and accounts for multiple comparisons when testing voxel by voxel [54]. Using this approach, we conducted a two sample t-test with variance smoothing ($\sigma = 6$mm) and 10000 permutations, to assess whether the voxel-wise balanced accuracies were significantly above the aforementioned empirical null distribution. Threshold-Free Cluster Enhancement [55] was used, and the family-wise error rate was controlled. This procedure yields a three-dimensional brain map of p-values, where each voxel indicates the probability that the balanced accuracy measured at said voxel across different subjects is statistically greater than chance. We chose a significance threshold of $\alpha =0.05$. Permutation testing offers a robust and assumption-free alternative in this setting, as opposed to the combination of t-tests with parametric corrections (such as FDR) at the cluster level, which can lead to inflated false positive rates [56].

We also used randomise to detect above-chance decoding for each subject independently, identifying informative clusters on an individualized basis. Although [54] advises against using this method on time-correlated data from the same subject, we can safely use it here because we are permuting accuracy values obtained in different data partitions with no time-series structure.

## Experiments

### Comparison of DA techniques

**Fig. 2:**
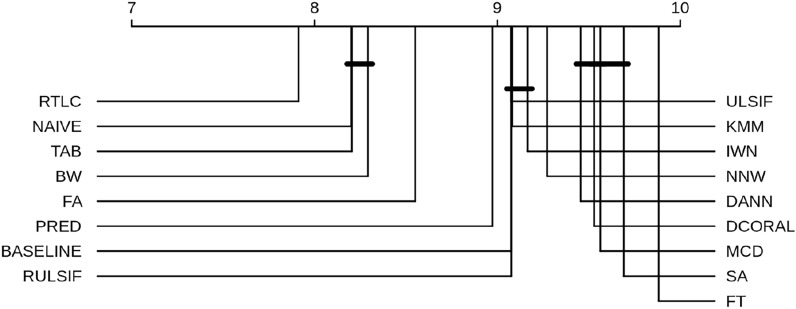
Critical Difference diagram. The scale above shows the average ranking of each algorithm across all observations, and the horizontal lines group together algorithms that are not statistically different

Figure 2 shows the unique Critical Difference (CD) diagram obtained by concatenating all the observations (pooling the results both across $N_t$ and across different subjects). The scale above shows the average ranking of each algorithm across all observations, and the horizontal lines group together algorithms that are not statistically different. From this, we conclude that RTLC was the best algorithm, followed by the group of NAIVE, TAB, BW and FA which are statistically indistinguishable ($\alpha =0.05$). On the other hand, FT is shown to be the worst.

**Fig. 3:**
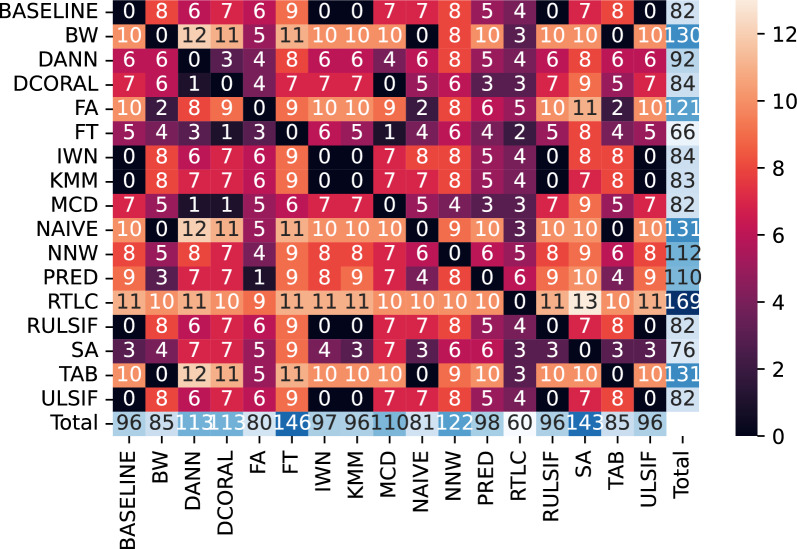
Frequency table. For each cell (*i*, *j*), the numerical value shows for how many subjects algorithm *i* was significantly superior to algorithm *j*. The sum of each row accounts for the number of times each algorithm was significantly better than others, and the sum of each column shows how many times each algorithm was significantly worse than others

Figure 3 summarizes the results of the tests in the 18 CD diagrams arising from concatenating the 10 tables corresponding to different $N_t$ values for each subject, which are shown in Supplementary Figure B2. For each cell (*i*, *j*) in this table, we report the number of subjects for which algorithm *i* was significantly better than algorithm *j* ($\alpha =0.05$). This can be extracted from the CD diagrams by counting how many times *i* appears to the left of *j* and subtracting the number of times they are joined by a horizontal line. The sum of each row represents the number of times each algorithm was better than others and, complementarily, the sum of each column reports the number of times each algorithm was significantly worse than others.

From Fig. 3, we extract the following highlights:

- RTLC outperformed others a total of 169 times, and was outperformed 60 times. It was the most outperforming and least outperformed algorithm.
- RTLC was better than BASELINE for 11 subjects, worse than BASELINE for 4 and statistically the same for the remaining 3.
- RTLC was better than NAIVE for 10 subjects, worse than NAIVE for 4 and statistically the same for the remaining 4.
- NAIVE was better than BASELINE for 10 subjects and worse for 7.
- FT was the most outperformed and least outperforming algorithm.

In the case of the “Generic Object Decoding” dataset, the best and worst methods were respectively PRED and DCORAL (Supplementary Figure B4). FA, BW and TAB were also better than NAIVE, although with no statistical significance. PRED outperformed other algorithms 47 times and was only outperformed once (Supplementary Figure B5). As detailed in B.2, the statistical power of the tests was diminished because of the lower number of subjects and feasible data partitions. Although no statistical significance was obtained, Supplementary Figure B6 reveals that FA and PRED were better than NAIVE in 4 subjects, BW for 3 and TAB for 2.

### Analysis of individual ROIs

**Fig. 4:**
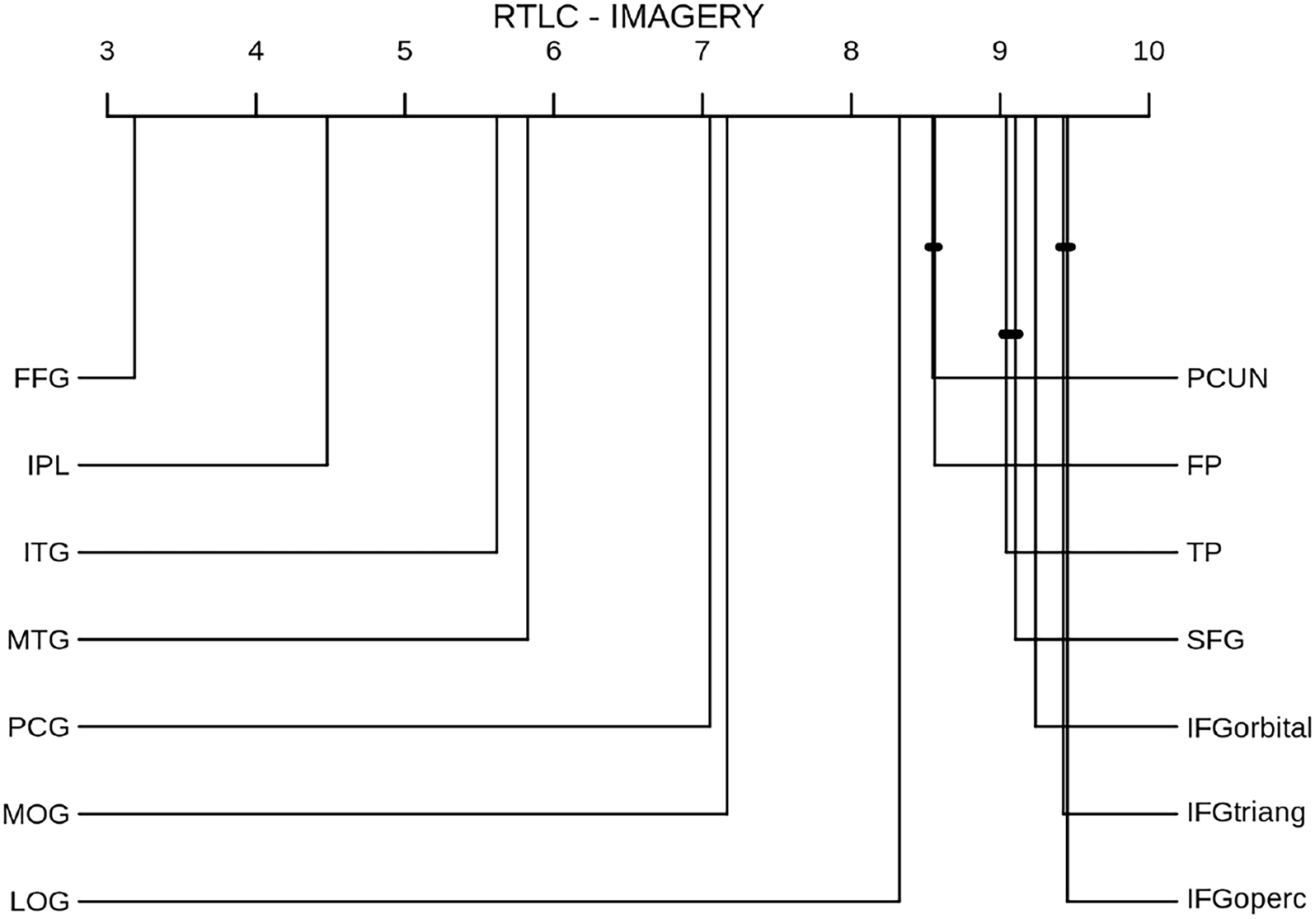
CD diagram of RTLC performance in the imagery domain across individual ROIs. FFG: Fusiform Gyrus; LOG: Lateral occipital gyrus; ITG: Inferior temporal gyrus; TP: Temporal pole; PCG: Posterior cyngulate gyrus; PCUN: Precuneus; IPL: Inferior parietal lobe; MTG: Middle temporal gyrus; SFG: Superior frontal gyrus; IFGoperc: Inferior frontal gyrus, pars opercularis, IFGtriang: Inferior frontal gyrus, pars triangularis; IFGorbital: Inferior frontal gyrus, pars orbitalis; FP: Frontopolar cortex; MOG: Medial orbital gyrus

Figure 4 shows the CD diagram of the RTLC performance in the imagery domain across individual ROIs, revealing the most informative regions for this method to be the fusiform gyrus (FFG), the inferior parietal lobe (IPL), the inferior temporal gyrus (ITG) and the middle temporal gyrus (MTG).

**Fig. 5:**
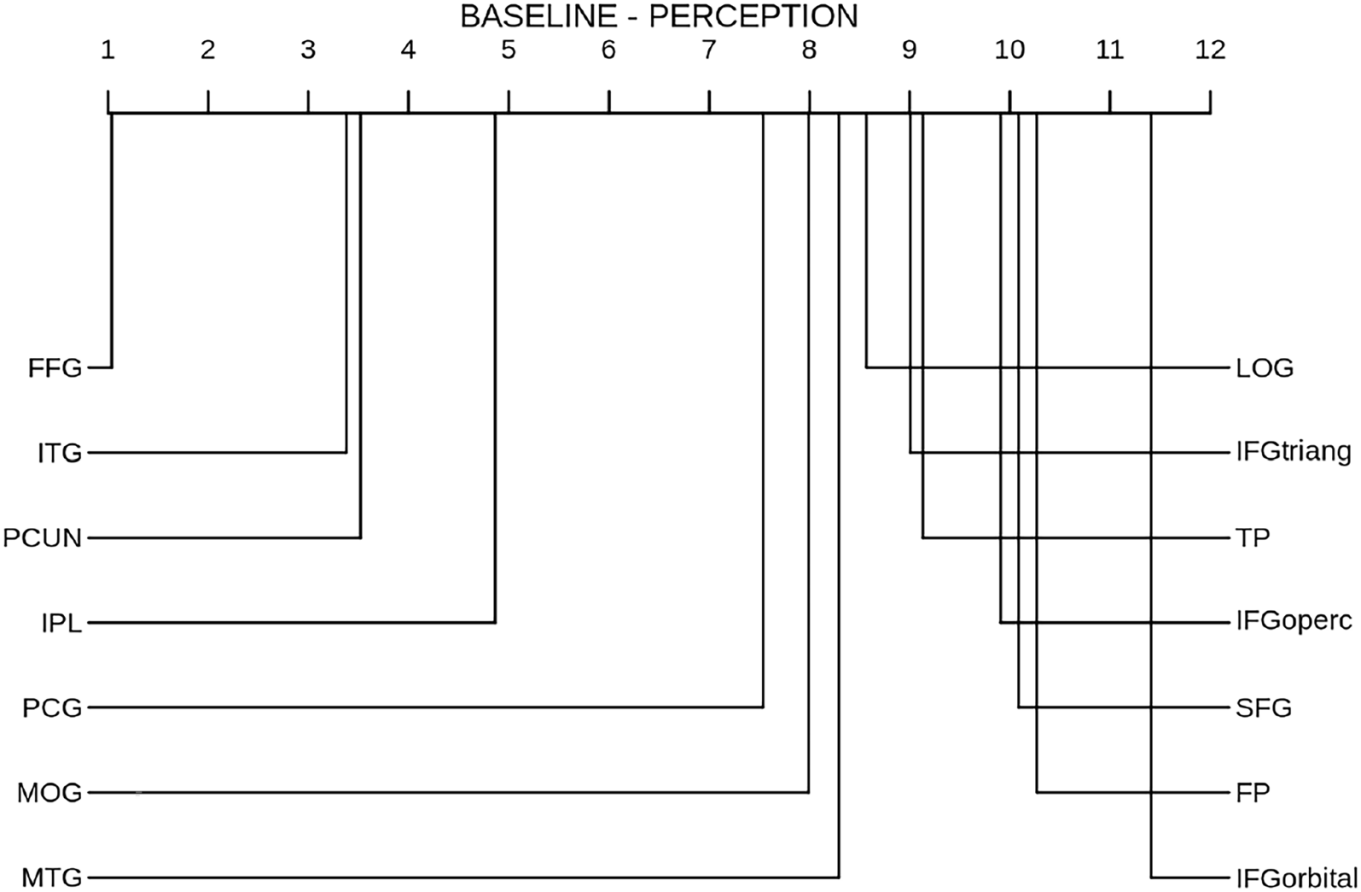
CD diagram of baseline performance in the perception domain across individual ROIs. FFG: Fusiform Gyrus; LOG: Lateral occipital gyrus; ITG: Inferior temporal gyrus; TP: Temporal pole; PCG: Posterior cyngulate gyrus; PCUN: Precuneus; IPL: Inferior parietal lobe; MTG: Middle temporal gyrus; SFG: Superior frontal gyrus; IFGoperc: Inferior frontal gyrus, pars opercularis, IFGtriang: Inferior frontal gyrus, pars triangularis; IFGorbital: Inferior frontal gyrus, pars orbitalis; FP: Frontopolar cortex; MOG: Medial orbital gyrus

Similarly, Fig. 5 highlights the fusiform gyrus as the most informative region in perception according to the baseline, with an average ranking of 1, meaning that it was the most informative region for all subjects and data partitions.

**Fig. 6:**
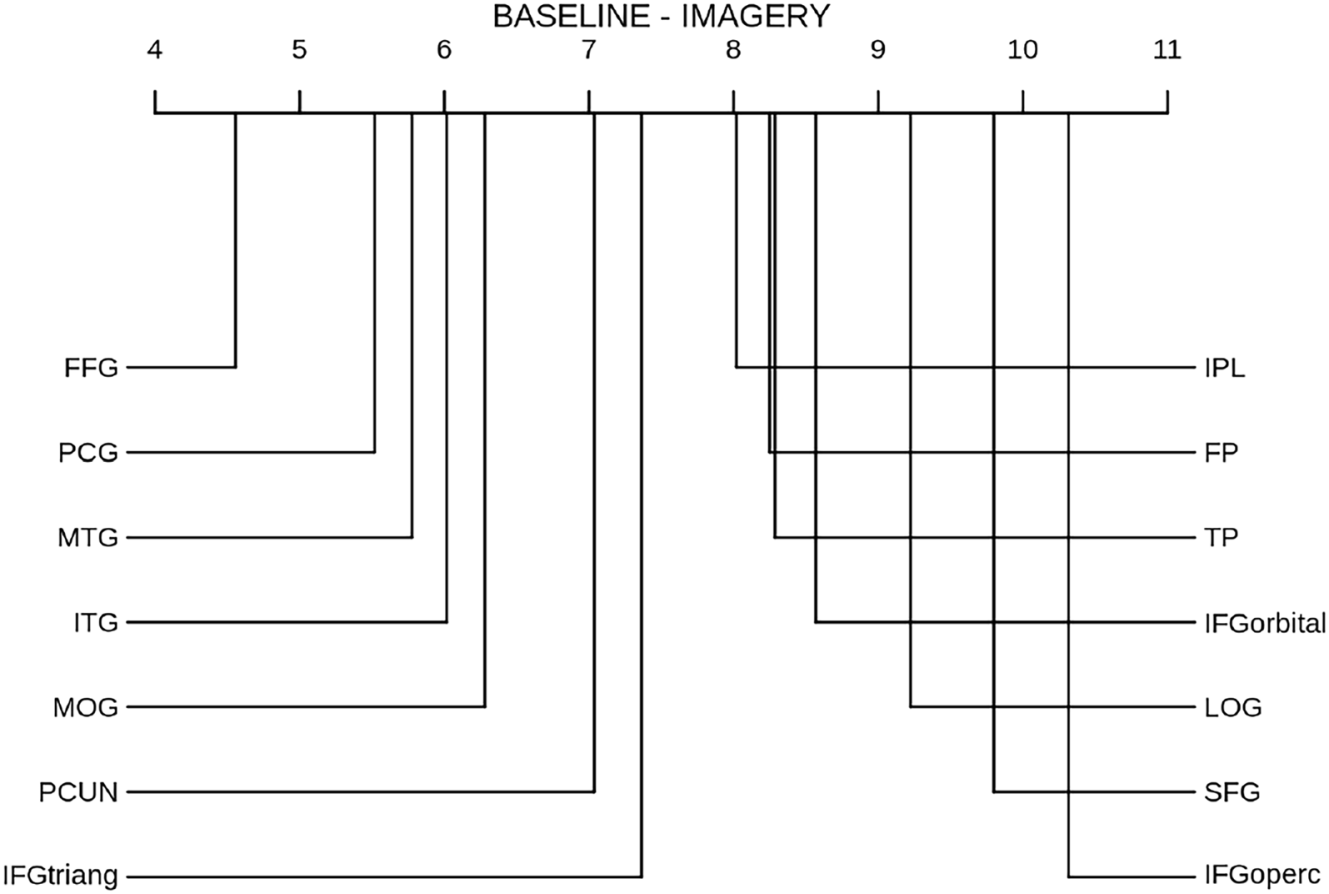
CD diagram of baseline performance in the imagery domain across individual ROIs. FFG: Fusiform Gyrus; LOG: Lateral occipital gyrus; ITG: Inferior temporal gyrus; TP: Temporal pole; PCG: Posterior cyngulate gyrus; PCUN: Precuneus; IPL: Inferior parietal lobe; MTG: Middle temporal gyrus; SFG: Superior frontal gyrus; IFGoperc: Inferior frontal gyrus, pars opercularis, IFGtriang: Inferior frontal gyrus, pars triangularis; IFGorbital: Inferior frontal gyrus, pars orbitalis; FP: Frontopolar cortex; MOG: Medial orbital gyrus

For completeness, Fig. 6 shows the ranking of ROIs acording to the baseline in the imagery domain. Although the fusiform gyrus remains the most informative ROI in this domain, its average ranking has increased, indicating subject variability in the imagery decoding across individual ROIs.

**Fig. 7:**
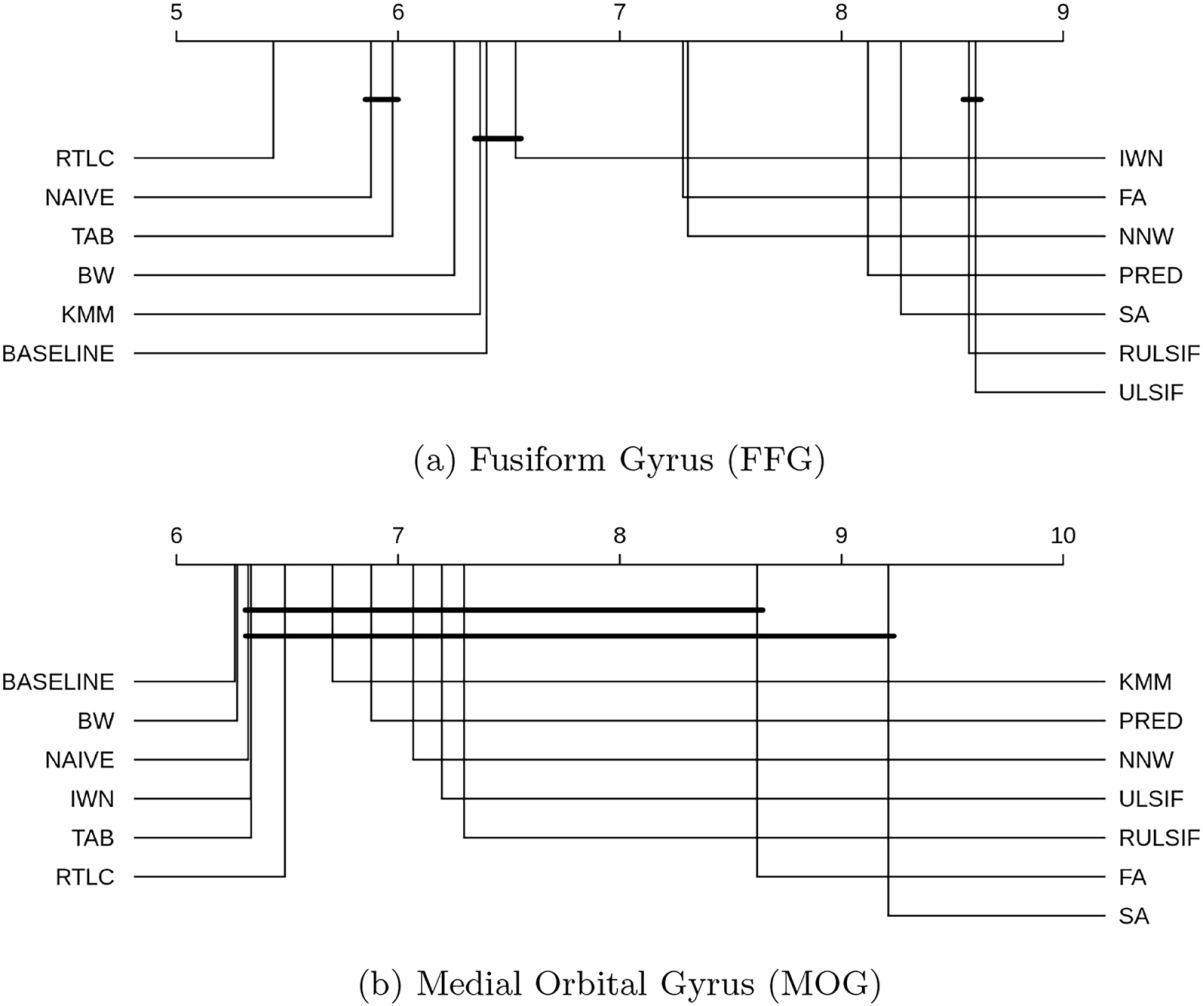
CD diagram of the DA comparison in a signal-rich region (FFG) versus a signal-poor control region (MOG)

As for the comparison of DA techniques in a task-relevant ROI and in a control ROI, the results are consistent with the whole-brain analyses. For the signal-rich region FFG, RTLC remains the best performing method (Fig. 7a). In contrast, in the MOG, all the approaches except the baseline and Balanced Weighting were statistically equal (Fig. 7b), suggesting that adaptation gains depend on the presence of task-relevant signal.

### DA-enhanced searchlight analysis

**Fig. 8:**
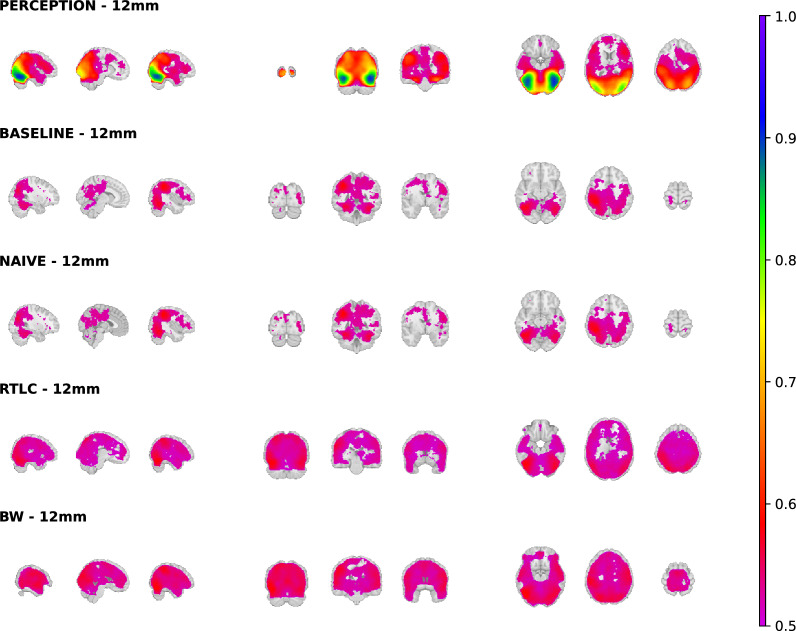
Between-subject average of the balanced accuracy of the different approaches in the regions that obtained statistically above chance decoding with RTLC

Figure 8 shows the projection of the average balanced accuracy of each approach (computed across the subjects) onto the areas of statistically significant brain decoding. We see that only within-domain prediction attains good balanced accuracy when averaged across all the subjects, and that in this case (perception) decoding is possible in a highly distributed set of brain regions. For the baseline and naïve approaches, decoding is only possible in a small, isolated region. On the contrary, our DA-enhanced searchlight with either RLTC and BW is able to obtain significantly above chance imagery decoding in an extensively spread brain volume.

**Fig. 9:**
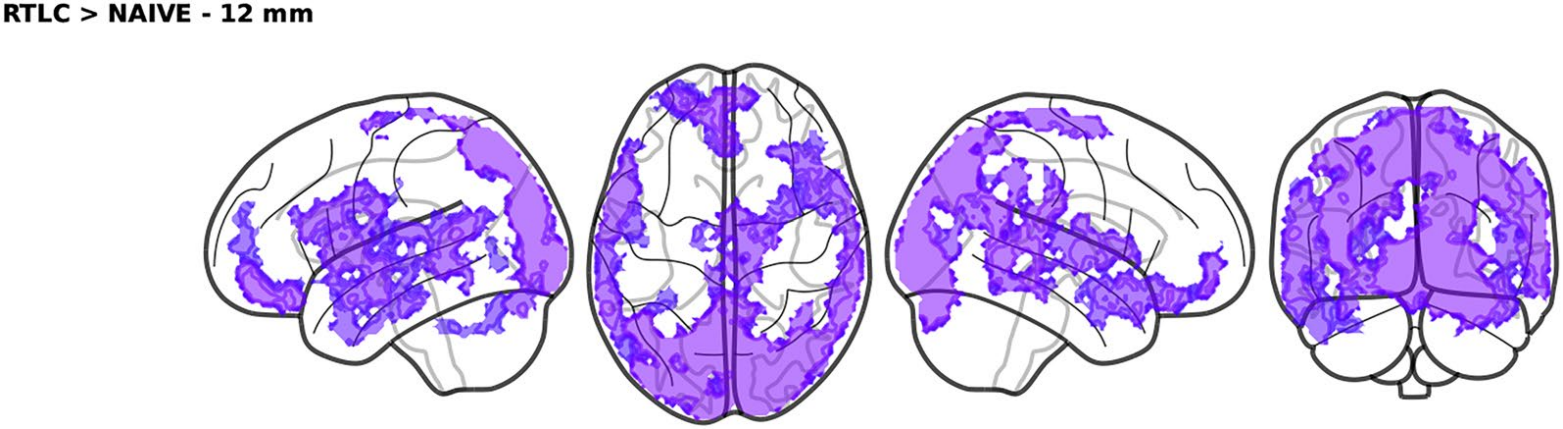
Brain regions where RTLC decoding is significantly better than the naïve approach ($p<0.05$, whole-brain corrected)

Figure 9 shows the results of the statistical comparison between RTLC and the naïve approach using a two-sample permutation test, highlighting only the statistically significant clusters ($p<0.05$, whole-brain corrected). The naïve method was never statistically significantly above the baseline. Supplementary Fig. C7 compares BW and naïve. Remarkably, a two-sample permutation test reveals that all the significant clusters that were found with RTLC are still present with BW, highlighting the robustness of the neuroscientific conclusions.

Moreover, Appendix C.3 shows that the results hold for different sizes of the searchlight sphere (9, 12 and 15 mm).

**Fig. 10:**
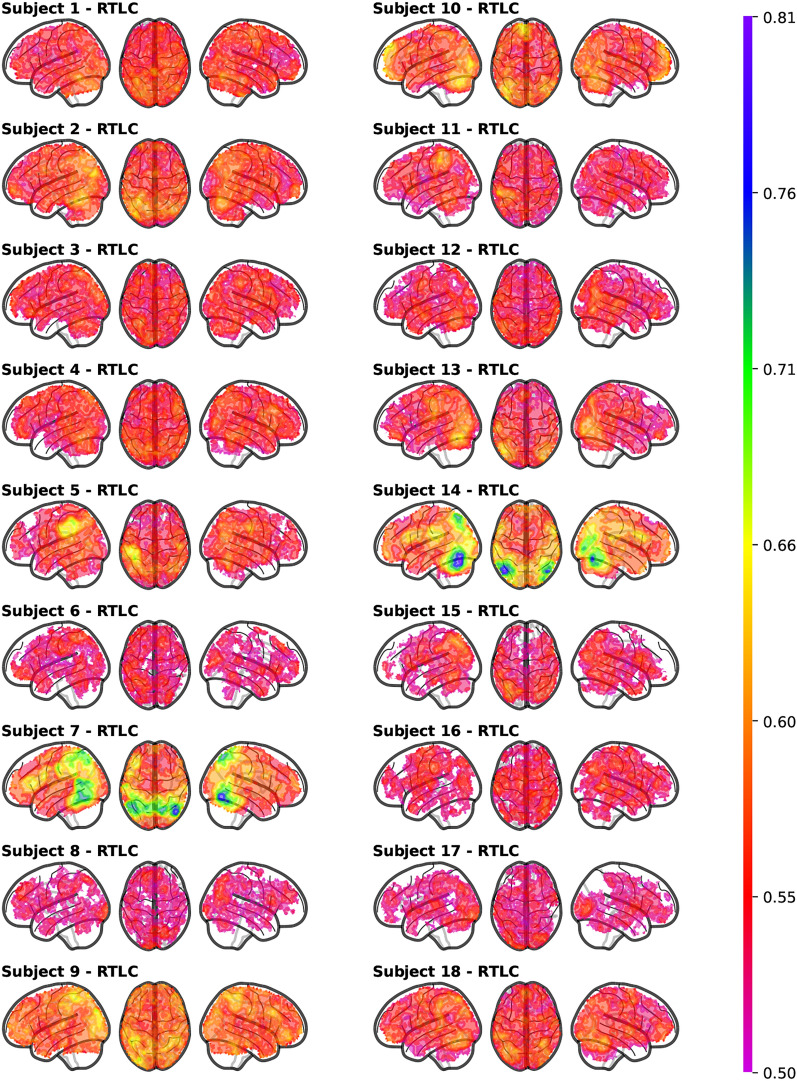
Inter-subject variability of the DA-enhanced searchlight accuracy. For each subject, the colour of each voxel represents the average RTLC accuracy over the 100 repetitions of the experiment in the regions where such accuracy is statistically above the voxel-wise empirical null distribution

Figure 10 accounts for the inter-subject variability of RTLC performance. Conversely, Supplementary Fig. C4 displays BW performance for each subject. Each set of brain slices corresponds to a different subject, and shows the average accuracy across the 100 data partitions projected onto the space where such average is statistically greater than chance level. Supplementary Figs. C1, C2 and C3, respectively, show the subject variability of the perception decoding, the baseline cross-domain decoding and the cross-domain decoding with naïve inclusion of target instances in the training set. Supplementary Figs. C5 and C6 show that the inter-subject variability observed in the results of the DA-enhanced searchlight does not exceed or contradict the one observed with standard searchlight procedures. Specifically, Supplementary Fig. C5 shows the pairwise correlation between individual subject accuracies across DA-enhanced searchlight methods (RTLC and BW) and the two standard searchlight procedures. Complementarily, Supplementary Fig. C6 displays the p-values of pairwise F-tests comparing the variance of the searchlight results across subjects and methods.

As illustrated in Supplementary Fig. C8c, the cross-subject average accuracy achieved using BW was higher compared to RTLC. Notably, with BW, our DA-enhanced searchlight successfully classifies imagery of Living versus Non-Living items in regions that extend into the frontal lobe. However, in terms of symmetry between the left and right hemispheres, the accuracy map for BW exhibits less balance compared to RTLC. This asymmetry is likely attributable to cross-subject averaging, as indicated by the comparison between Fig. 10 and Supplementary Fig. C4. Specifically, BW demonstrates poor decoding performance for subjects 6, 9, and 17, whereas RTLC achieves successful decoding for these individuals.

## Discussion

Many neuroscience studies face challenges due to distribution shifts, but most cross-domain experiments in cognitive neuroscience do not address this, which can be mitigated with DA techniques. DA does not directly align voxels between conditions, but instead reveals whether a learnable transformation can support generalization across domains. If successful, DA suggests the presence of shared high-level representations encoded differently across conditions, which are inaccessible through standard decoding. Excluding DA from cross-condition studies limits the discovery of shared neural representations and reduces the applicability of classifiers trained on one domain to others.

For example, in decoded neurofeedback studies, classifiers decode a participant’s cognitive state in real time and provide feedback to induce a specific activation pattern [57]. While several studies have used imagery or motor task decoding [46, 58–60], none incorporate DA to improve feedback accuracy.

Our results support the hypothesis that DA can enhance predictive models trained with fMRI data from different conditions both at the ROI level and in searchlight analyses, aligning with prior work showing DA benefits for BCIs [61, 62]. Importantly, DA methods outperform naïve addition of target data in conventional classifiers. RTLC improved decoding on the binary dataset, and PRED was best in the multiclass setting. Additionally, 6 out of 16 DA methods significantly outperformed the baseline in the binary dataset (Fig. 2), and 8 methods outperformed it in the multiclass dataset (Supplementary Fig. B4).

BW and TAB were equivalent to NAIVE in the ROI comparison, suggesting that these methods may perform better in local analyses, where the curse of dimensionality is less of an issue. Indeed, BW’s DA-enhanced searchlight analysis outperforms NAIVE, while TAB is not suitable for the searchlight due to its iterative nature and high computational cost.

In addition to enhancing classifier transferability, the success of different DA methods in our study could provide clues into the nature of the domain shifts between perception and imagery tasks in fMRI data. Each method’s assumptions and mechanisms offer a lens through which we can interpret their effectiveness. For instance, RTLC addresses both covariate and concept shift, penalizing the deviation of the target model parameters from those of the source model while fitting to target data. This assumes that, although the target domain may need a tailored classifier, the source model contains structure that is still informative. Its success in our binary dataset, encompassing 14 ROIs across the brain, suggests that the transformation from perception to imagery in a whole-brain level involves linear shifts in decision boundaries. This implies that the underlying neural representations for these tasks are distinct but linearly related, supporting the notion of shared high-level representations that are differently expressed across conditions. For the “Generic Object Decoding” dataset, the best techniques were FA and PRED. FA assumes that some features behave differently in the source and target domains (domain-specific components), while others behave similarly (domain-invariant or general components). By categorizing the features into those three groups, the method is primarily addressing the shift in the feature distribution *P*(*X*) while learning the decision function *P*(*Y*|*X*) jointly. PRED, on the other hand, addresses both covariate and concept shifts. It captures this by incorporating the predictions from a source-trained model as an explicit new feature for the target classifier. This implies that the mapping from features to labels *P*(*Y*|*X*) may differ between source and target (i.e., concept shift), but the source model may still carry informative structure that can help the target model.

Our DA-enhanced searchlight approach revealed a significant generalization improvement between perception and imagery in the visual cortex and beyond, in frontoparietal regions.

Assessing DA’s effect on predictive ability provides deeper insight into brain representations. Improved classification indicates that initial distribution differences in the data hinder the baseline model’s ability to use all information. This predictive improvement is crucial for applications like neurofeedback, where accurate decoding is essential.

DA success is constrained by the signal-to-noise ratio of the data. Poor baseline models in the source domain result in weak generalization to the target domain. For instance, not all ROIs contribute valuable information for visual object category decoding, as shown in Fig. 5. The FFG is the most informative ROI for all subjects, while regions like the IFGorbital, FP, and SFG encode minimal information.

While perception-to-perception decoding is reliable in occipital regions, not all subjects show extended visual information in other brain regions (Supplementary Fig. C1 - perception).

For imagery, the baseline models fail to decode effectively, as seen in Fig. 8 (baseline and naïve). Subject-specific variability in domain shift (Supplementary Figs. C2 and C3) reveals that some participants, like Subject 7 and Subject 14, exhibit good out-of-the-box transferability, while others, like Subject 5, show sparse decoding regions. In a group-level, the average decoding is confined to small brain areas, making standard models unreliable for unseen imagery data. DA-enhanced models, however, achieve above-chance decoding in the same areas as perception.

We also performed a sensitivity analysis on the searchlight radius, using 9, 12 and 15 mm. Supplementary Figs. C9a and C9b confirm that statistical significance clusters are consistent across sphere sizes. Supplementary Fig. C8 shows that decoding accuracy is not strongly dependent on the radius for any of the algorithms.

Regarding why RTLC and PRED outperform other DA methods in the binary and multiclass datasets respectively in the ROI-based comparison, the simplicity of these techniques might make them adequate to deal with high-dimensional and noisy data.

RTLC offers distinct advantages in the binary dataset, where multiple ROIs were combined. Feature-based methods rely on global transformations of the input space, but fMRI data often exhibit heterogeneous domain shifts across regions, especially when multiple ROIs are combined. In such cases, a global feature alignment may be suboptimal. Instance-based methods aim to adjust the influence of individual training samples from the source domain based on their similarity to the target distribution, but obtaining robust similarity estimates is notoriously difficult in high-dimensional fMRI data. In turn, RTLC adapts the classifier parameters directly. This approach reduces the risk of overfitting in high-dimensional spaces where sample sizes are limited, and allows accommodating region-specific variations more effectively.

This interpretation is supported by our findings: RTLC outperformed other methods in the binary dataset that spans the whole brain but underperformed in the multiclass dataset restricted to the visual cortex – a functionally coherent region likely characterized by a more homogeneous shift. In that scenario, feature-based methods that assume global consistency across features, such as FA and PRED, performed better.

The BW technique also performed remarkably in the DA-enhanced searchlight, despite not attaining a statistically significant improvement over NAIVE at the ROI level. There are, in fact, some similarities between the methodology of RTLC and BW. BW works by fitting $h_{DA}$ directly on source and target labelled data according to the modified loss $(1-\gamma )\mathcal {L}(h(X_s),y_s)+\gamma \mathcal {L}(h(X_t),y_t)$, where the parameter $\gamma$ controls the trade-off between fitting the source or the target data. In a way, this is related to RTLC because it minimizes a combined source-target loss where the target component can also be considered a penalty term, although RTLC does it in two consecutive steps.

From the point of view of ML, it is important to analyse the limitations of current DA techniques in real-world scenarios. Usually, DA methods are tested considering artificial benchmarks such as Office-31 [63] and MNIST [64] or real problems with a low dimensionality. fMRI data offers the opportunity to evaluate these methods, elucidating whether the complexity of the methods is translated into an enhanced performance. Therefore, one of the goals of this paper is to evaluate the performance of state-of-the-art DA techniques when dealing with high-dimensional, noisy and complex data.

From a more practical perspective, establishing an accurate learner that is capable to generalize from perception to imagery is relevant for many neuroscience applications, including but not limited to BCIs, neurofeedback training, and clinical scenarios. For instance, in rehabilitation settings, decoding brain activity patterns associated with mental imagery and visual perception could be used to develop interventions for individuals with visual impairments or neurological disorders affecting visual processing.

Among the limitations of our work is the lack of a comprehensive characterization of the 18 participants, which was not feasible with the available data. Our results show considerable inter-subject variability in both baseline classifier performance and DA effectiveness. While this is expected due to the individuality of brain processes, we cannot fully account for it. For instance, subjective imagery vividness – as assessed in [65] – could have clarified how individual strategies impact DA improvements. Unfortunately, this information was unavailable for our dataset.

Another limitation concerns the comparison of DA methods. Some techniques that performed poorly at the ROI level might have outperformed RTLC in the searchlight analysis, which is less affected by the curse of dimensionality. This was the case for BW, which slightly surpassed RTLC in the DA-enhanced searchlight. However, due to the high computational demands of fitting tens of thousands of models per data split, we were unable to extend voxel-wise comparisons to all DA methods. While RTLC and BW were the only techniques broadly tested in the searchlight, we acknowledge that they are not necessarily optimal in this context. More complex methods, such as neural networks, are impractical for searchlight use due to their computational cost. However, the overlap between the clusters where either RTLC or BW were significantly better than NAIVE highlights the robustness of our results.

We also note the lack of suitable alternative datasets to reinforce our neuroscientific claims. Although we demonstrated DA’s benefit for perception-to-imagery generalization in a public multiclass dataset, this dataset is unsuited for searchlight analysis. It includes only 5 subjects, limiting statistical power for pairwise comparisons, and lacks the transformation matrices needed for aligning searchlight results to a common space (e.g., MNI).

From a cognitive neuroscience perspective, our study explores shared mechanisms between perception and imagery – an area with limited available data. However, the DA-enhanced searchlight method introduced here is general and can be applied to other domain-shift problems in neuroscience. Future work could explore distribution shifts in high-dimensional fMRI, identify subject-level factors that predict DA success, and apply DA in real-time clinical contexts like neurofeedback. For such applications, DA must be fast, robust, and user-specific; developing lightweight, online DA techniques is a key step toward clinical deployment.

Currently, inter-individual variability in brain structure and function remains a major obstacle to model generalization. This is especially critical in subjective tasks such as imagery. Although cross-subject DA is beyond our current scope, extending our method to this setting is a promising direction for future research.

Notably, our results emphasize the strong subject-dependency of imagery processes, which show far more inter-subject variability than perception (see Supplementary Figs. C1 and C2). By analyzing each subject independently, we gained insight into individual decoding profiles (Supplementary Figs. 10 and C2). Through MNI transformation and permutation-based testing, we also derived generalizable group-level insights for our study population.

## Conclusions

In this paper, we have introduced an effective way to integrate DA into the searchlight procedure, significantly enhancing classification performance in cross-domain brain decoding.

Through a comprehensive analysis involving data from 14 ROIs and 18 subjects for a classification task distinguishing between living and non-living objects, we demonstrated that several DA techniques outperformed our baseline model. The baseline model was trained solely on perception data and tested on imagery data. Similar results were observed on a publicly available multiclass dataset, highlighting a clear domain shift between neural activities associated with visual perception and imagery.

The best performing method in our dataset was RTLC. Utilizing RTLC, we assessed both the nature and location of the domain shift within our DA-enhanced searchlight approach. Remarkably, RTLC enabled above-chance decoding of imagined stimuli in a highly distributed set of brain regions, including the visual cortex and the frontoparietal cortex. This contrasts sharply with non-DA searchlight procedures, which did not achieve comparable performance. To strengthen the robustness of our neuroscientific findings, we replicated the results using a DA-enhanced searchlight approach with an alternative DA technique (BW).

These findings are particularly promising for applications in BCIs and neurofeedback training, suggesting that DA techniques like RTLC can substantially improve the decoding of imagined stimuli or any other critical brain state that investigators may seek to induce in decoded neurofeedback studies. This advancement paves the way for more effective and reliable cross-domain brain decoding methods, potentially revolutionizing how neural data is utilized in practical applications.

## Supplementary Information

Additional file 1.

## Data Availability

The binary perception/imagery dataset analysed during the current study is available in the Open Science Framework (OSF) repository, https://osf.io/audmx/. The “Generic Object Decoding” dataset that supports the findings of this study is available in Figshare with the identifier https://figshare.com/articles/dataset/Generic_Object_Decoding/7387130. The code for this paper is available at the GitHub repository AlexOlza/DA-enhanced-searchlight.

## Abbreviations

DA: Domain Adaptation
MRI: Magnetic resonance imaging
fMRI: Functional MRI
MVPA: Multivoxel (or multivariate) pattern classification analysis
BCI: Brain-computer interface
ROI: Region of interest
ML: Machine learning
EEG: Electroencephalography
CNN: Convolutional neural network
MNI: Montreal neurological institute
LR: Logistic regression
CD: Critical difference (diagram)
FFG: Fusiform gyrus
IPL: Inferior parietal lobe
ITG: Inferior temporal lobe
MTG: Middle temporal gyrus
