# Supplementary material for: Domain Adaptation-enhanced searchlight: enabling classification of brain states from visual perception to mental imagery
Source: Brain Inform. 2025 Jun 28;12(1):17. doi: 10.1186/s40708-025-00263-0 (PMC12206218; doi:10.1186/s40708-025-00263-0)
Supplement: Supplementary file 1 — Additional file 1. [file 40708_2025_263_MOESM1_ESM.pdf]

# Appendix A
## Supplementary Results

### Appendix A.1 Additional information about the fMRI datasets

| Abbreviation | Name |
|---|---|
| FFG | Fusiform gyrus |
| LOG | Lateral occipital gyrus |
| ITG | Inferior temporal gyrus |
| TP | Temporal pole |
| PCG | Posterior cingulate gyrus |
| PCUN | Precuneus |
| IPL | Inferior parietal lobe |
| MTG | Middle temporal gyrus |
| SFG | Superior frontal gyrus |
| IFGoperc | Inferior frontal gyrus, pars opercularis |
| IFGorbital | Inferior frontal gyrus, pars orbitalis |
| IFGtriang | Inferior frontal gyrus, pars triangularis |
| FP | Frontopolar cortex |
| MOG | Medial orbital gyrus |

**Table A1**: Abbreviations and names of the ROIs under study

| | | | Perception | | Imagery | |
|---|---|---|---|---|---|---|
| Subject | Voxels (ROIs) | Voxels | Instances | Prevalence | Instances | Prevalence |
| 1 | 10373 | 35744 | 368 | 0.503 | 234 | 0.513 |
| 2 | 10675 | 35464 | 362 | 0.500 | 233 | 0.494 |
| 3 | 10383 | 37775 | 362 | 0.494 | 232 | 0.509 |
| 4 | 10852 | 41513 | 362 | 0.517 | 233 | 0.511 |
| 5 | 10768 | 40194 | 362 | 0.506 | 233 | 0.494 |
| 6 | 10577 | 41162 | 364 | 0.495 | 233 | 0.498 |
| 7 | 11078 | 47814 | 364 | 0.500 | 231 | 0.511 |
| 8 | 11178 | 43787 | 366 | 0.511 | 233 | 0.502 |
| 9 | 13916 | 51748 | 361 | 0.504 | 234 | 0.491 |
| 10 | 11841 | 46884 | 362 | 0.497 | 231 | 0.506 |
| 11 | 10807 | 41465 | 362 | 0.503 | 233 | 0.511 |
| 12 | 12171 | 44995 | 364 | 0.500 | 232 | 0.496 |
| 13 | 12208 | 46920 | 365 | 0.496 | 233 | 0.506 |
| 14 | 10823 | 38441 | 365 | 0.496 | 234 | 0.500 |
| 15 | 12127 | 40111 | 360 | 0.500 | 234 | 0.491 |
| 16 | 9161 | 33991 | 364 | 0.489 | 232 | 0.500 |
| 17 | 12179 | 45649 | 363 | 0.510 | 233 | 0.511 |
| 18 | 11214 | 43522 | 361 | 0.507 | 234 | 0.504 |

**Table A2**: Dataset characteristics for each subject

| Dataset | Source | | Target | |
|---|---|---|---|---|
| | Train | Test | Train | Test |
| Binary | 44-46 | 10-12 | 26-28 | 8-10 |
| GOD (Semantic) | 26-28 | 7-9 | 14-15 | 5-6 |
| GOD (ResNet) | 26-29 | 6-9 | 13-15 | 5-7 |

**Table A3**: Number of trials in train/test partitions.

## Appendix A.2 Brief description of each DA method used

- `BalancedWeighting` [20]: A supervised, instance-based method that fits the base estimator $h$ source and target labelled data according to the modified loss $(1-\gamma)\mathcal{L}(h(X_s), y_s) + \gamma\mathcal{L}(h(X_t), y_t)$, where the parameter $\gamma$ controls the ratio between fitting the source data and adapting to the target data, with higher values indicating more adaptation. In our case, $\mathcal{L}$ is the binary cross-entropy and $\gamma = 0.5$. `BalancedWeighting` can be used with any supervised estimator.
- `RegularTransferLC` (Regular Transfer for Linear Classification - RTLC) [32]: A supervised, parameter-based method that starts from a source-only parametric linear classifier and translates the classification problem in the target domain into a linear regression task, minimizing the Mean Squared Error with a penalty on the Euclidean distance of the $h_{DA}$ parameters and the source-only model parameters. In short, given a source-only linear model with parameters $\beta_s$, it constructs a linear model for the target domain with parameters $\beta_t = \underset{\beta}{argmin}||X_t\beta - y_t||^2 + \lambda||\beta - \beta_s||$. Higher $\lambda$ indicates a stronger fit to the available target instances, while lower values involve a higher use of the source domain knowledge.
- `KMM` (Kernel Mean Matching) [23]: An unsupervised, instance-based sample bias correction approach that minimizes the Maximum Mean Discrepancy (MMD) between source and target domains by solving a quadratic optimization problem such that the means of $X_s$ and $X_t$ in a reproducing kernel Hilbert space (RKHS) are closer than a predefined threshold. We used the default Radial Basis Function kernel.
- `TrAdaBoost` (Transfer AdaBoost for Classification) [21]: A supervised, instance-based iterative reweighing approach. Firstly, an initial estimator is fitted on the concatenation of labelled source and target data. Then, the samples are reweighed based on the performance of said estimator, decreasing the weight of the wrongly classified source instances under the hypothesis that those instances are more dissimilar to the ones coming from the target domain.
- `FA` [26]: A supervised, feature-based pre-processing step leveraging feature augmentation, which aims to separate features into (a) those specific to the source domain, (b) those specific to the target domain and (c) those common to both, without altering the model fitting stage, so it can be applied to any supervised estimator.
- `PRED` [26]: Another feature-augmentation DA technique, mixing the idea in `FA` with the notion of model stacking. First, the base estimator is trained solely on source data, predicting target values on the available target data. These predictions are

concatenated with $X_t$ as a new feature, and a new instance of the estimator is trained on the resulting data-set to obtain the final $h_{DA}$.

- `ULSIF` (Unconstrained Least-Squares Importance Fitting) [24]: An unsupervised, instance-based method to correct the difference between input distributions of source and target domains as measured by their relative Pearson divergences by reweighing the source instances. This entails solving a quadratic optimization problem, which has a closed-form solution involving the inverse of a matrix $H$ of size equal to the number of samples in $X_s$. This matrix contains the element-wise products of a kernel transformation applied to $X_s$. $H^{-1}$ is then multiplied with the kernel transformation of $X_t$ to give the weights of the source samples. We used an RBF kernel.
- `NearestNeighborsWeighting` (NNW) [66]: An unsupervised, instance-based approach that computes, for each source domain sample, the number of neighbours inside a certain radius in the target data set. Source instances are reweighted proportionately.
- `RULSIF` (Relative Unconstrained Least-Squares Importance Fitting) [25]: A very similar approach to ULSIF, but allowing the target instances to influence the matrix $H$ via an additive contribution of the element-wise products of their kernel transformation.
- `SA` (Subspace Alignment) [28]: An unsupervised, feature-based method that linearly aligns the source domain to the target domain in a reduced PCA subspace of a certain pre-specified dimension.
- `IWN` (Importance Weighting Network) [22]: An unsupervised, instance-based method that reweighs the source domain samples minimizing the MMD between the reweighed source and the target distributions by using a neural network to predict the sample weights, thus reducing the computational burden of KMM.
- `FineTuning` (FT) [27]: A feature-based, supervised DA method originally developed for Convolutional Neural Networks, that reuses the internal layers of a network trained in the source domain and furthers training of the external layers using a limited amount of target domain data.
- `DeepCORAL` (Deep CORrelation ALignment - DCORAL) [31]: Correlation Alignment is a feature-based and unsupervised DA method that aligns the second-order statistics of the source and target distributions using a linear transformation. Similarly, Deep CORAL is a nonlinear extension that aligns correlations of layer activations in deep neural networks.
- `DANN` (Discriminative Adversarial Neural Network) [30]: DANN is a feature-based DA method, which strives to find a new representation of the input features in which source and target data are indistinguishable by any discriminator network. This new representation is given by an encoder which is learned along the discriminator using a reversal gradient layer. In parallel, a task network is learned on the encoded space.
- `MCD` (Maximum Classifier Discrepancy) [29]: MCD is a feature-based, unsupervised DA method that looks for a new representation of the input features which minimizes the discrepancy between the source and target domains. The discrepancy is

computed through adversarial training of three networks: An encoder and two classifiers, each of which learn the task on the source and the target domains. A reversal layer is placed between the encoder and the two classifiers to perform adversarial training.

# Appendix A.3 Details on data acquisition

## A.3.1 Binary dataset

The dataset includes fMRI scans of 18 untrained participants who took part in visual perception and imagery tasks. The complete data acquisition pipeline is described in [46].

The subjects completed 4 image presentation runs. Each run was composed of 14 trials with a duration of 22s and an inter-trial interval (ITI) of 8500ms. Each trial consisted in the visual presentation of 12 different images from the same category, interleaved with 250ms visual fixation periods. The same 12 images of each category were used in all trials, but they were presented in random order.

In the imagery experiment, participants received auditory cues indicating the target category and were instructed to visualize such category for 12 seconds. The auditory prompt was repeated three times during the 12s period. The experiment spanned two imagery runs, each of which comprised nine trials per category.

To match the characteristics of the hemodynamic response function (HRF), the authors selected the fMRI volumes occurring between 5 and 18s following the initial picture/auditory prompt, yielding seven volumes per trial.

## A.3.2 "Generic Object Decoding"

This dataset contains fMRI scans of 5 healthy subjects with considerable experience in fMRI experiments. The participants completed a visual perception and an imagery task. The full data acquisition procedure is described in [34].

The image presentation experiment consisted of 35 runs (9min 54s each). Each run contained 55 stimulus blocks. Subjects performed a one-back repetition detection task on the images to maintain their attention. A total of 50 images from 50 object categories (1 image per category) were presented 35 times each in randomized order.

In the imagery experiment, subjects were required to visually imagine images from 1 of the 50 categories that were presented in the test image session of the image presentation experiment. The imagery experiment consisted of 20 runs and each run contained 25 imagery blocks (10min 39s per run). Each imagery block included a 3-s cue period and a 15-s imagery period. The imagery cues consisted of the written name of the target category. The 25 categories in each run were pseudo-randomly selected from the 50 categories in the perception experiment.

The normalized voxel amplitudes from each experiment were averaged within each 9-s stimulus block (image presentation experiment) or within each 15-s imagery period (imagery experiment), after shifting the data by 3s (one volume) to compensate for hemodynamic delays.

# Appendix B
## Supplementary results on the DA comparison

### Appendix B.1 Binary dataset

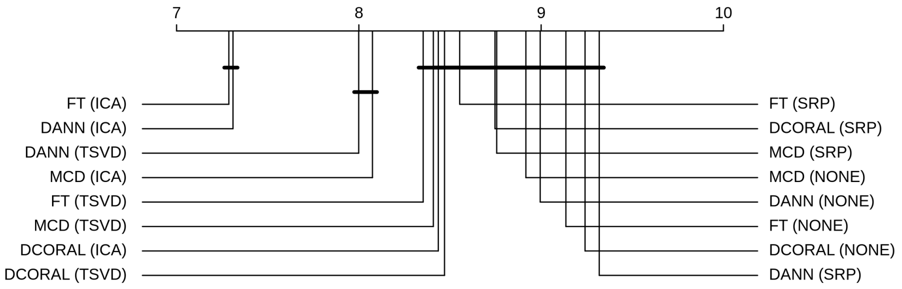


**Fig. B1**: CD diagram comparing the effect of different dimensionality reduction techniques in the Deep Learning DA methods. NONE: Raw features; SRP: Sparse Random Projection; ICA: Independent component analysis; TSVD: Truncated Singular Value Decomposition.

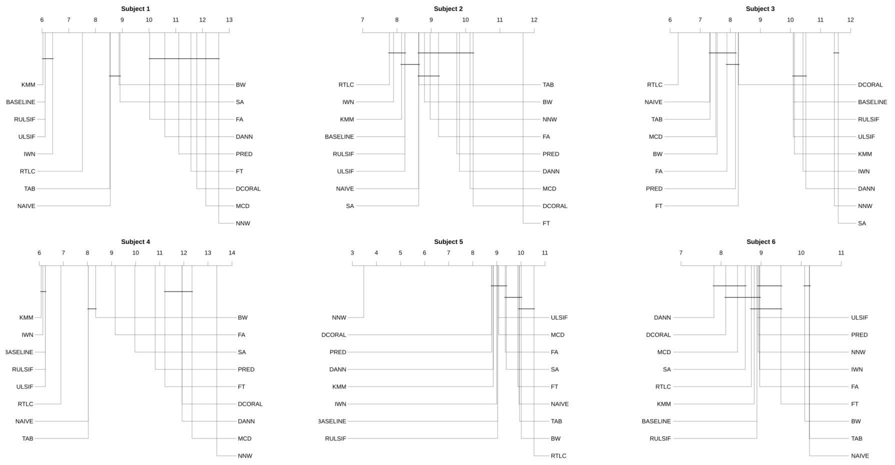


**Fig. B2**: Critical Difference diagrams for each subject, comparing different DA techniques on the union of 14 ROIs (Part 1).

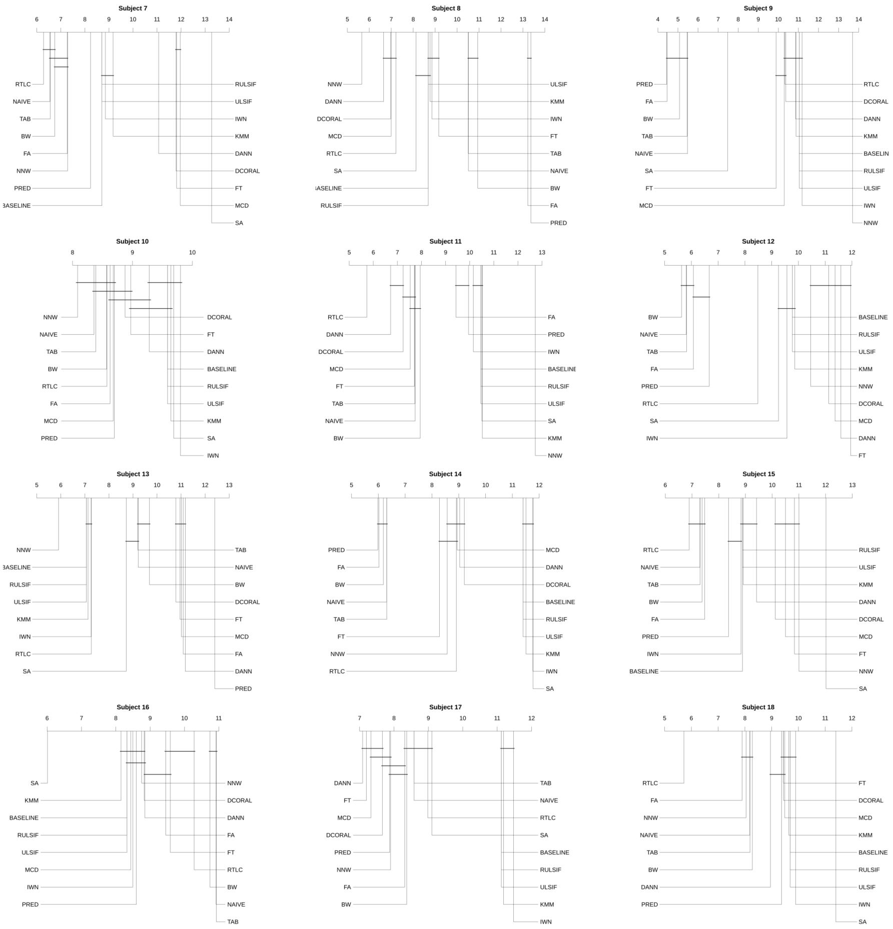


**Fig. B2**: Critical Difference diagrams for each subject, comparing different DA techniques on the union of 14 ROIs (continued).

# Appendix B.2 Results on "Generic Object Decoding" dataset

## B.2.1 Dataset description

The publicly available "Generic Object Decoding" dataset [34] gathers fMRI scans from 5 healthy subjects who were exposed to visual stimuli from 50 different categories (perception condition) and were later instructed to imagine those categories (imagery condition). Both of these experiments constituted the evaluation data for the methodology proposed in the original paper [34]. Data acquisition and preprocessing are discussed in [34].

In the perception condition, each category was presented a total of 35 times and the experiment involved 35 runs. The stimuli presentation order was randomized across runs. Each category included a single representative image, which was extracted from the THINGS [48] database.

The imagery condition involved 20 runs, each of which included half of the categories in a semi-random order, such that every two consecutive runs contained all the categories. Each run was composed of 25 imagery events, and therefore, each category was imagined a total of 10 times during the experiment.

This dataset allows the opportunity to test the applicability of DA methods to assess the transferability of visual perception decoding to the imagery domain in a multiclass classification setting. However, the reduced amount of imagery data per class (10 examples) is a key limitation for training and evaluation, both of which require all categories to be present in the corresponding data partitions. Furthermore, the need to ensure that examples from the same experimental run are not present in both train and test partitions renders training impossible given the distribution of categories across the runs in the data acquisition step.

For the reasons listed above, investigating the impact of DA on this dataset is only feasible if the number of classes is reduced in a way that preserves the correlation between class labels and neural activity. We have conducted the comparison of DA methods on this dataset using two different methods to encode the class labels: On one hand, a semantic grouping yielding 9 broader classes (Table B4), one of which must be discarded due to insufficient imagery data ("Weapons", containing only the subclass "Cannon": 10 examples). This semantic grouping results in an imbalanced dataset, because each broader class contains a different number of subclasses. Many other semantic groupings are possible, as long as they are crafted in such a way that preserves the human interpretation of the stimuli. On an independent experiment, we have encoded the labels using a data-driven approach grounded in computer vision: We have extracted representative embeddings for each class using a pretrained ResNet model, and performed hierarchical clustering on these embeddings to group the classes based on their feature representation. We used cluster quality measures to

select the number of clusters (three). See next section for a detailed explanation, and Supplementary Table B5 for the resulting groups of labels.

| Broad Category | Original Subclasses | Number of Examples |
|---|---|---|
| Animals | Goldfish, Owl, Common iguana, Duck, Swan, Conch, Crab, Killer whale, Leopard, Bat, Housefly, Butterfly, Goat, Camel, Domestic llama | 150 |
| Clothing and Accessories | Cowboy hat, Silk hat, Football helmet, Sock, Welder's mask | 50 |
| Household Items | Beer mug, Bowling ball, Hammock, Mailbox, Umbrella | 50 |
| Musical Instruments | Electric guitar, Grand piano, Harp, Mandolin, Tambourine | 50 |
| Structures and Monuments | Coffin, Gravestone, Minaret, Stained-glass window | 40 |
| Technology and Devices | IPod, Microwave, Videocassette recorder, Washing machine | 40 |
| Tools and Equipment | Fire extinguisher, Knob, Tweezer, Planchet, Shredder | 50 |
| Vehicles and Transportation | Airliner, Barrow, Bulldozer, Canoe, Covered wagon, Snowmobile | 60 |
| Weapons | Cannon | 10 |

**Table B4**: Broad categories, their original subclasses, and the number of examples.

| Broad Category | Original Subclasses | Number of Examples |
|---|---|---|
| Cluster 1 | Beer mug, Bowling ball, Cowboy hat, Silk hat, Electric guitar, Fire extinguisher, Football helmet, iPod, Knob, Mandolin, Tweezer, Planchet, Shredder, Sock, Tambourine, Videocassette recorder, Washing machine, Welder's mask | 190 |
| Cluster 2 | Airliner, Barrow, Bulldozer, Cannon, Coffin, Covered wagon, Grand piano, Gravestone, Hammock, Harp, Mailbox, Minaret Snowmobile, Stained-glass window, Umbrella | 160 |
| Cluster 3 | Goldfish, Owl, Common iguana, Duck, Swan, Conch, Crab' Killer whale, Leopard, Bat, Housefly, Butterfly, Goat, Camel, Domestic llama, Canoe | 150 |

**Table B5**: Broad categories, their original subclasses, and the number of examples.

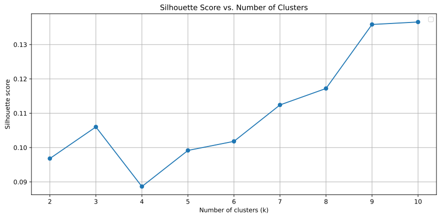


(a) Silhouette scores for varying number of clusters

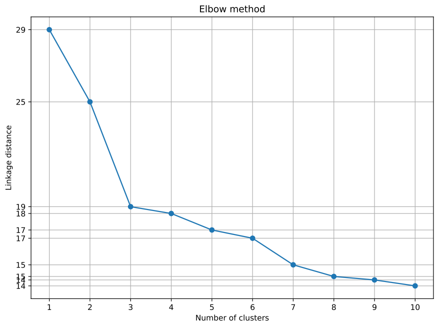


(b) The elbow method

**Fig. B3**: Cluster quality measures for the ResNet-based class grouping.

## B.2.2 Methodology

### B.2.2.1 ResNet-based class groupings

Given that the stimuli presented to participants during the acquisition of this fMRI dataset were natural images extracted from ImageNet, we used a computer vision-based approach to group the classes based on their visual attributes.

Using a pre-trained ResNet50 [67], we computed the last layer embeddings of 200 images from each of the 50 classes present in the GOD dataset. The dimension of the vector embedding was 2048. Then, we averaged the embeddings within each class, obtaining 50 embedding vectors, each of which serves as a class representative.

Finally, we used hierarchical clustering with Ward linkage to cluster similar embeddings, and hence similar classes (as interpreted by the ResNet model). Contrary to the grouping proposed in B4, in this case the notion of similarity is not semantic or functional, but is instead based on deep computer vision.

We use the silhouette score (Supplementary Figure B3a) and the elbow method (Supplementary Figure B3b) to select the number of clusters, and we obtain 3 final categories shown in Table B5.

### B.2.2.2 Validation framework

We performed the comparison of the 15 DA methods under study, as well as the NAIVE and BASELINE methods as defined in Section 4. The validation framework was exactly the same, except for the number of data partitions which, with the semantic class grouping, decreased from 100 to 24 as a consequence of requiring all 8 classes to be present for both training and testing. The ResNet-based class grouping allowed for 100 iterations. For both class groupings, the number of target domain instances in the training set ($N_t$) was also increased to account for the multiclass setting ($N_t = 200, 250, 300$). Random over-sampling was used in both the source and

target domains to balance the dataset before training.

We used all the voxels from the largest ROI provided by the dataset authors, which covers the Visual Cortex (VC) as defined in [34] Supplementary Figure 1. Because of the reduced number of subjects and viable data partitions, the total experiments per algorithm were significantly fewer for the “Generic Object Decoding” dataset, impacting the power of the statistical tests.

### B.2.3 Results

#### B.2.3.1 Semantic class grouping

From Figure B4 we conclude that PRED, FA, BW and TAB were the best algorithms. Among those, PRED was statistically significantly better than NAIVE ($p < 0.05$).

Figure B5 indicates that PRED significantly outperformed other algorithms 47 times and was only outperformed once (by NNW). It was the most outperforming algorithm along with BW. Similarly, FA, BW and TAB were outperformed only once (by NNW).

Due to the decrease in the number of subjects and feasible data partitions, the total number of experiments per algorithm was dramatically lower for the “Generic Object Decoding” dataset, which affects the power of the statistical tests summarized in figures B4 and B5. Figure B6 shows the results for each subject, revealing that FA, PRED and BW were better than NAIVE in 4 subjects and TAB for 2.

Regarding class-wise performance, Figure B7 shows the confusion matrices for the baseline, NAIVE and PRED algorithms, taking all subjects and iterations into account.

Finally, Figure B8 shows the performance ranking of the Deep Learning techniques with different dimensionality reduction techniques and also with the raw features.

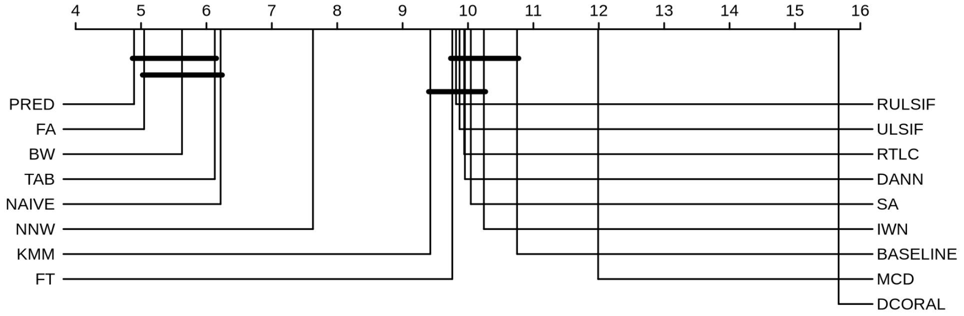


**Fig. B4**: Critical Difference diagram. The scale above shows the average ranking of each algorithm across all observations, and the horizontal lines group together algorithms that are not statistically different.

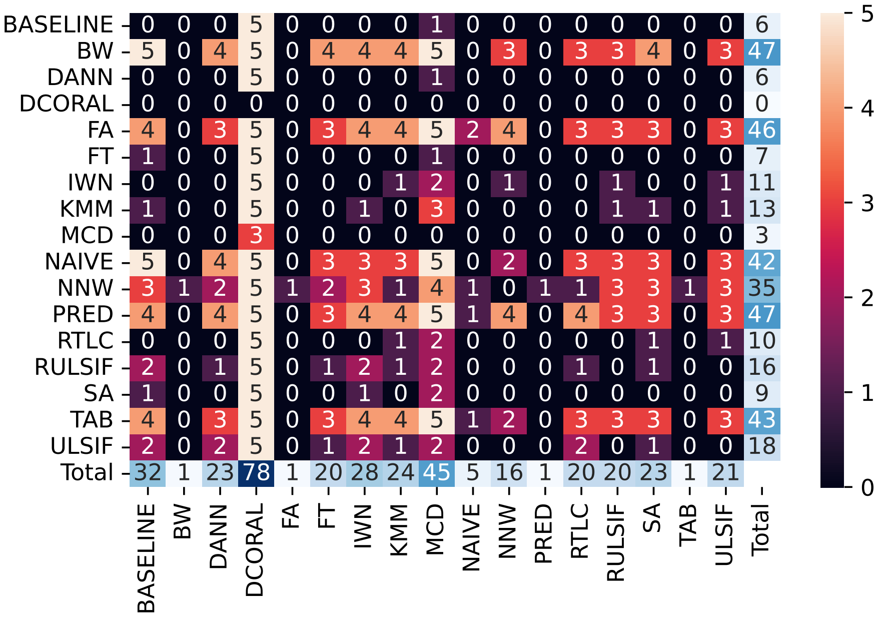


**Fig. B5**: Frequency table. For each cell $(i, j)$, the numerical value shows for how many subjects algorithm $i$ was significantly superior to algorithm $j$. The sum of each row accounts for the number of times each algorithm was significantly better than others, and the sum of each column shows how many times each algorithm was significantly worse than others.

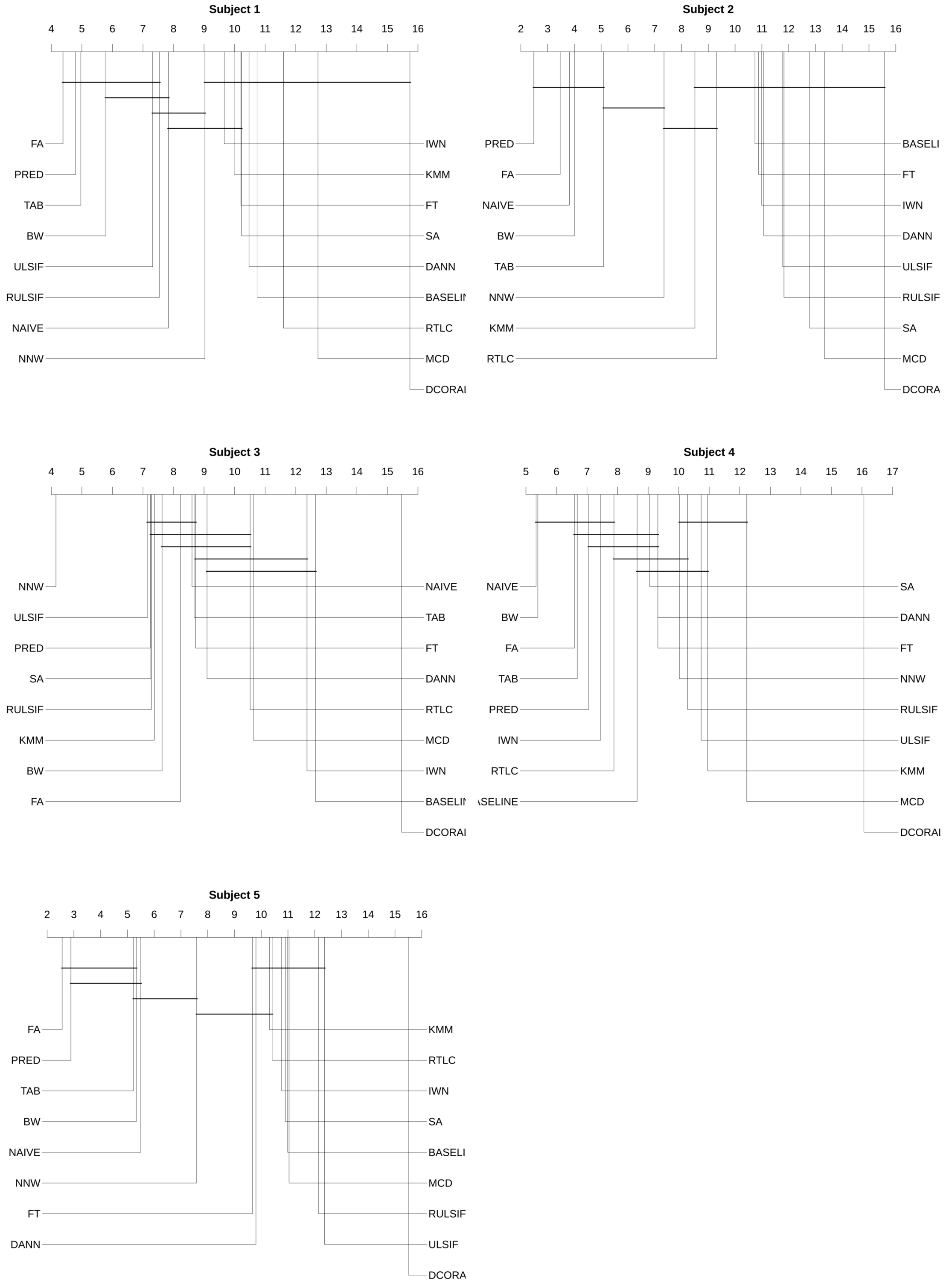


**Fig. B6**: Critical Difference diagrams for each subject, comparing different DA techniques in the Visual Cortex.

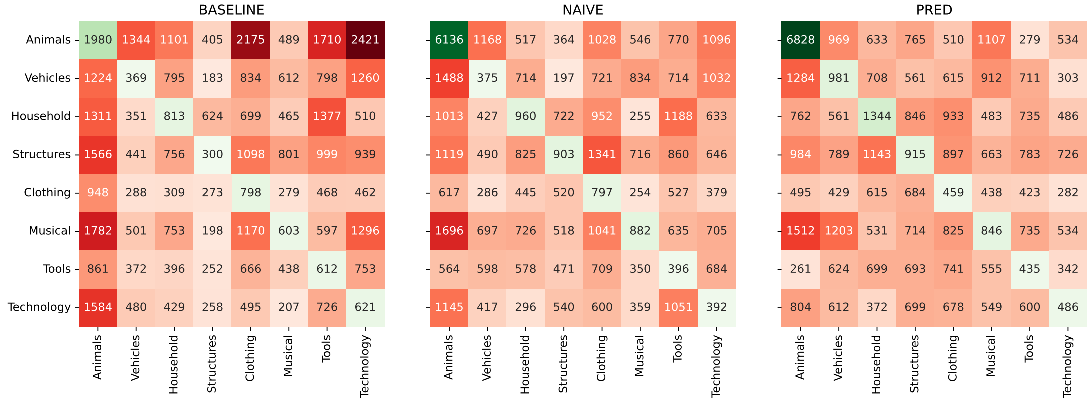


**Fig. B7**: Confusion matrix for the baseline, NAIVE and PRED methods, taking into account the imagery test set predictions and true labels for all the subjects and train-test splits iterations.

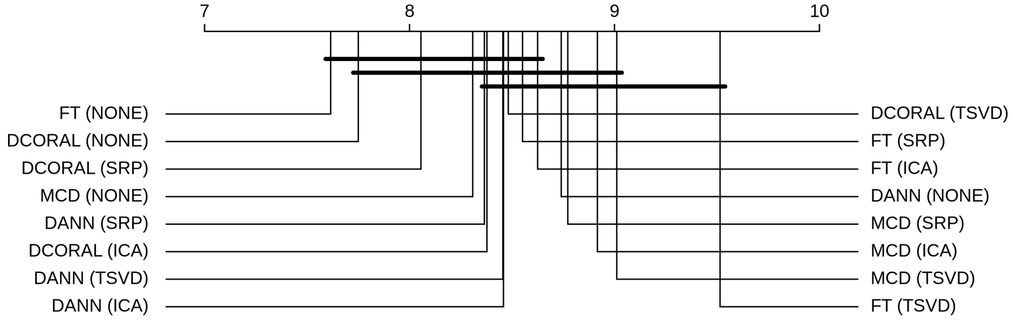


**Fig. B8**: CD diagram comparing the effect of different dimensionality reduction techniques in the Deep Learning DA methods. NONE: Raw features; SRP: Sparse Random Projection; ICA: Independent component analysis; TSVD: Truncated Singular Value Decomposition.

### B.2.3.2 ResNet-based class grouping

From Figure B9 we conclude that PRED, FA, BW, and TAB were the best algorithms. Among those, PRED was statistically significantly better than NAIVE ($p < 0.05$).

Figure B10 indicates that FA and PRED significantly outperformed other algorithms 65 times, and were only outperformed 3 and 4 times respectively. They were the most outperforming algorithms.

Figure B11 shows the results for each subject, revealing that FA was superior to NAIVE for all subjects, PRED and TAB for 4 subjects, and BW for 3.

Figure B12 shows the confusion matrices for the baseline, NAIVE and PRED algorithms, considering all subjects and iterations.

Finally, Figure B13 shows the performance ranking of Deep Learning techniques with different dimensionality reduction techniques and also with the raw features.

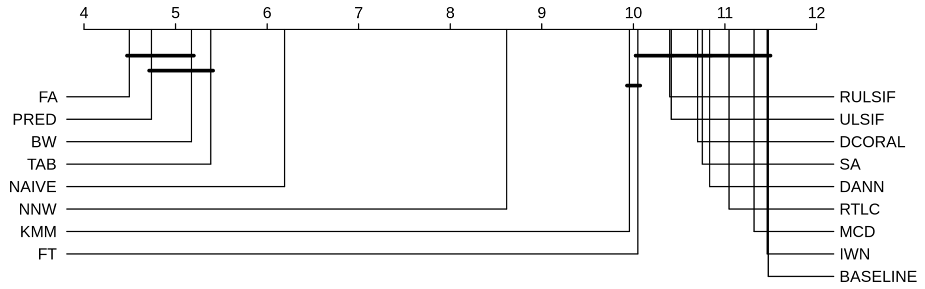


**Fig. B9**: Critical Difference diagram. The scale above shows the average ranking of each algorithm across all observations, and the horizontal lines group together algorithms that are not statistically different.

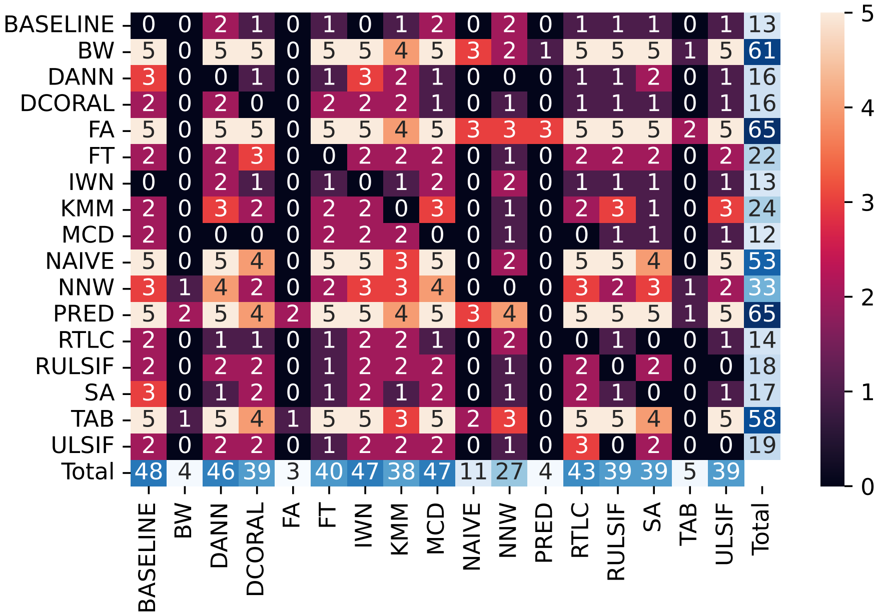


**Fig. B10**: Frequency table. For each cell $(i, j)$, the numerical value shows for how many subjects algorithm $i$ was significantly superior to algorithm $j$. The sum of each row accounts for the number of times each algorithm was significantly better than others, and the sum of each column shows how many times each algorithm was significantly worse than others.

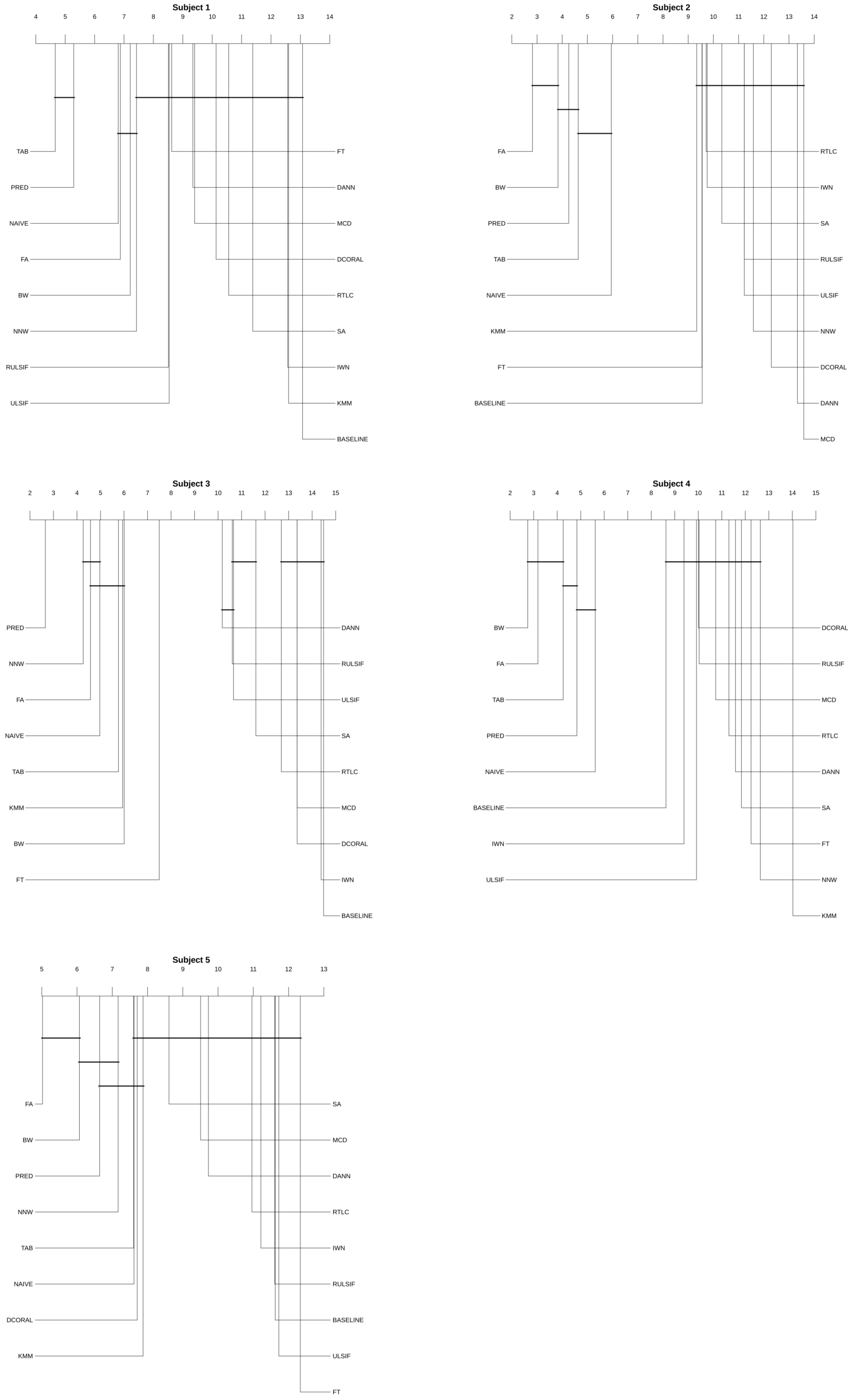


**Fig. B11**: Critical Difference diagrams for each subject, comparing different DA techniques in the Visual Cortex.

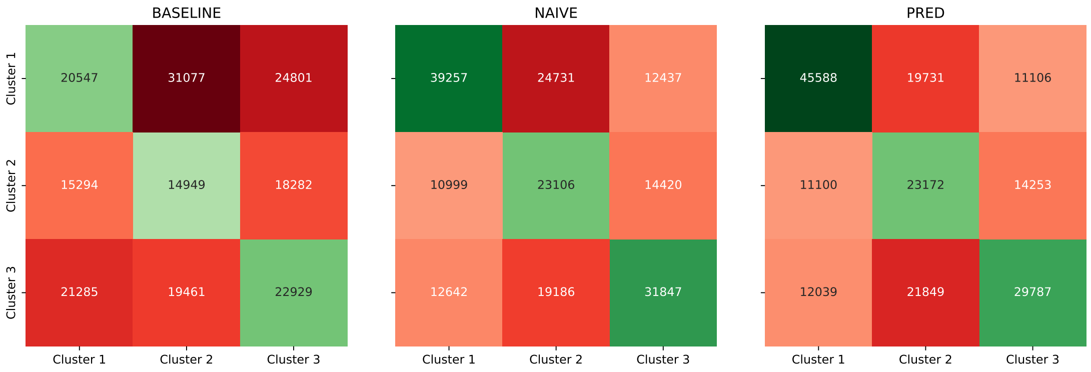


**Fig. B12**: Confusion matrix for the baseline, NAIVE and PRED methods, taking into account the imagery test set predictions and true labels for all the subjects and train-test splits iterations.

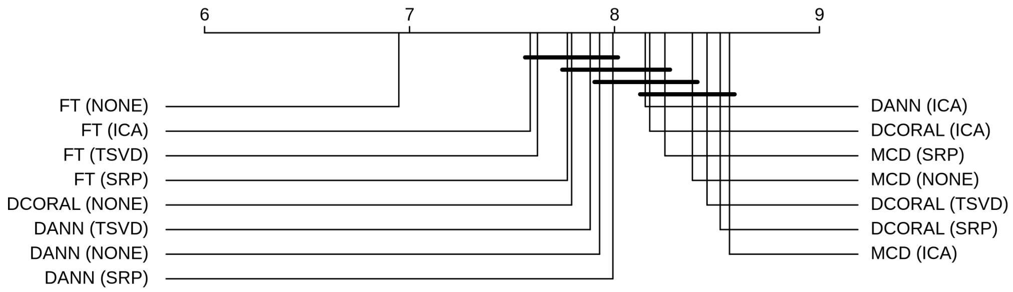


**Fig. B13**: CD diagram comparing the effect of different dimensionality reduction techniques in the Deep Learning DA methods. NONE: Raw features; SRP: Sparse Random Projection; ICA: Independent component analysis; TSVD: Truncated Singular Value Decomposition.

# Appendix C
## Supplementary results on the DA-enhanced searchlight

### Appendix C.1 Subject variability

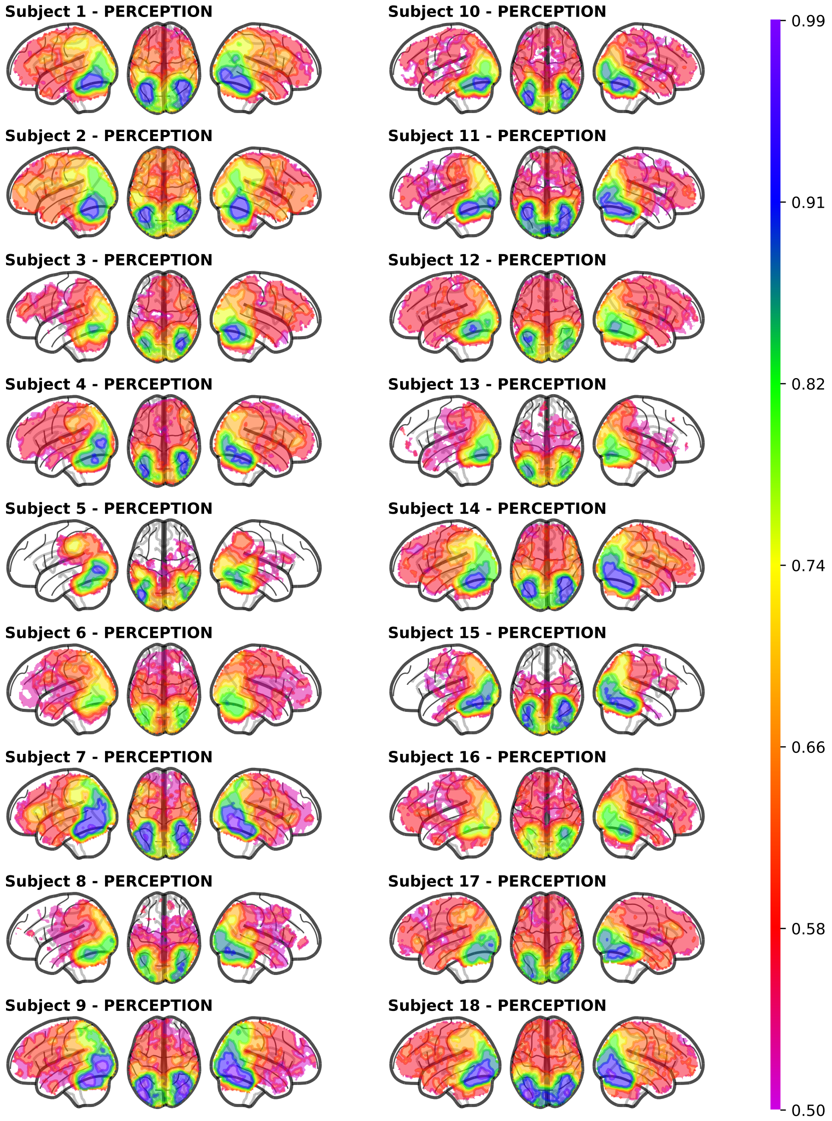


**Fig. C1**: Subject variability of the within-domain searchlight accuracy. For each subject, the colour of each voxel represents the average perception-to-perception accuracy over the 100 repetitions of the experiment in the regions where such accuracy is statistically significantly above the voxel-wise empirical null distribution.

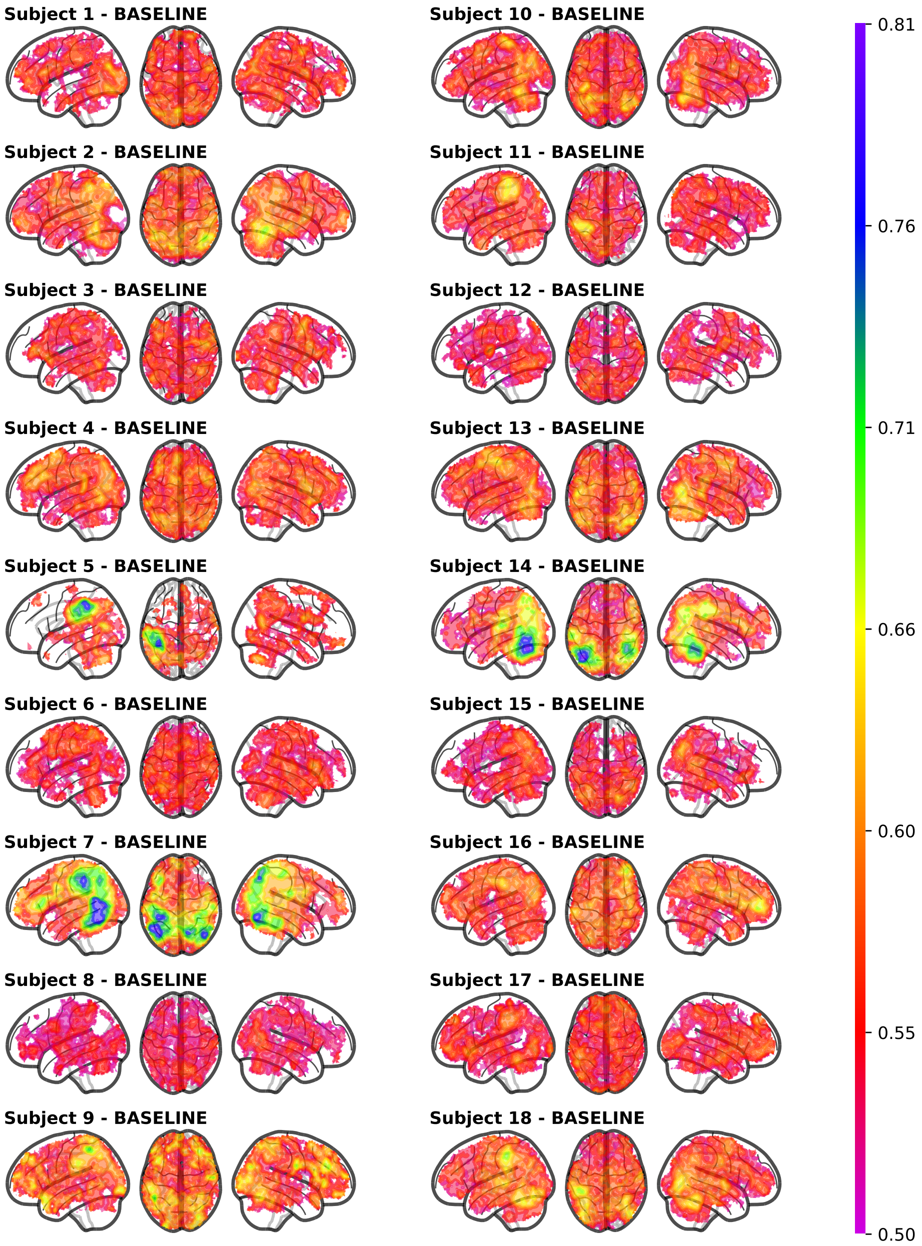


**Fig. C2**: Subject variability of the cross-domain searchlight accuracy. For each subject, the colour of each voxel represents the average baseline perception-to-imagery accuracy over the 100 repetitions of the experiment in the regions where such accuracy is statistically significantly above the voxel-wise empirical null distribution.

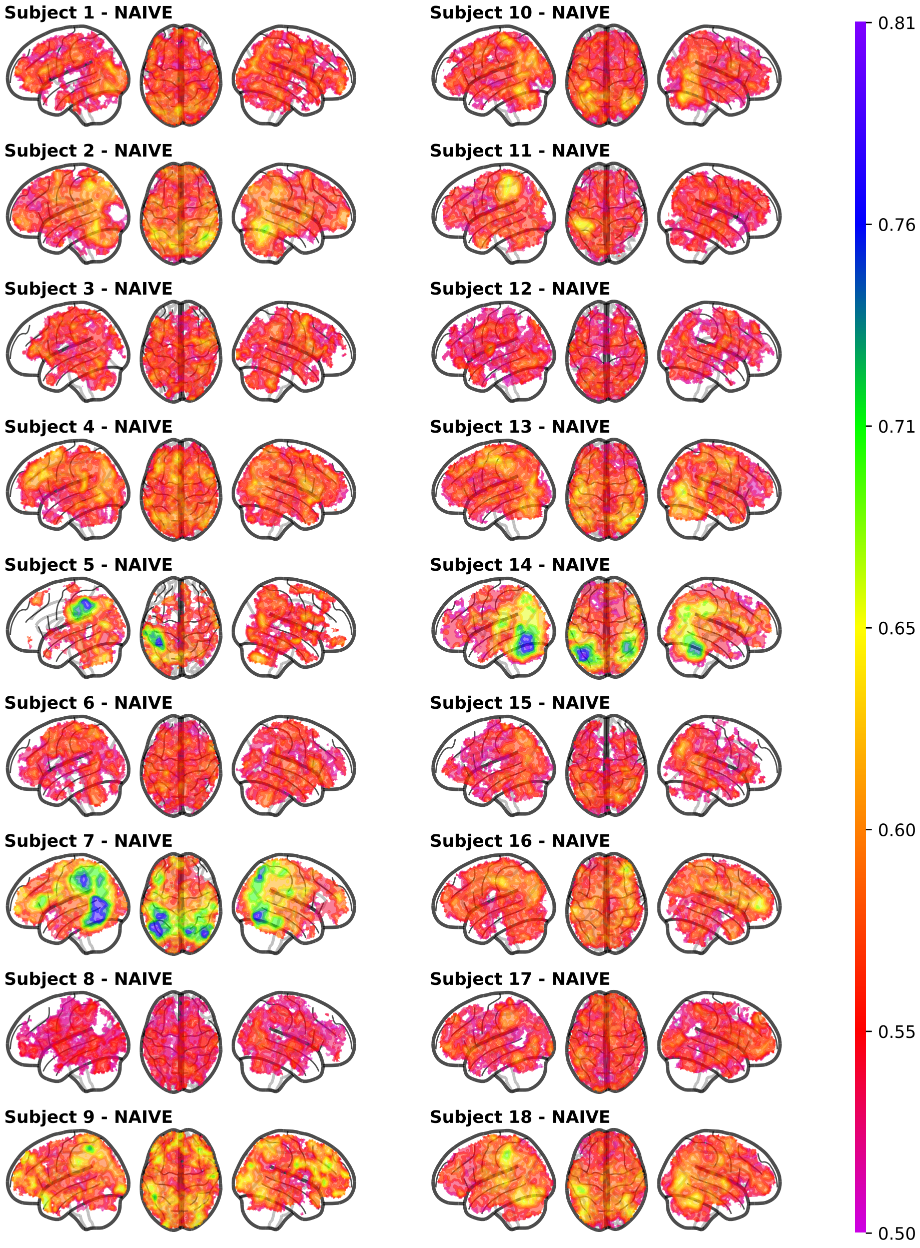


**Fig. C3**: Subject variability of the cross-domain searchlight accuracy with 100 target domain instances available during training. For each subject, the colour of each voxel represents the average accuracy over the 100 repetitions of the experiment in the regions where such accuracy is statistically significantly above the voxel-wise empirical null distribution.

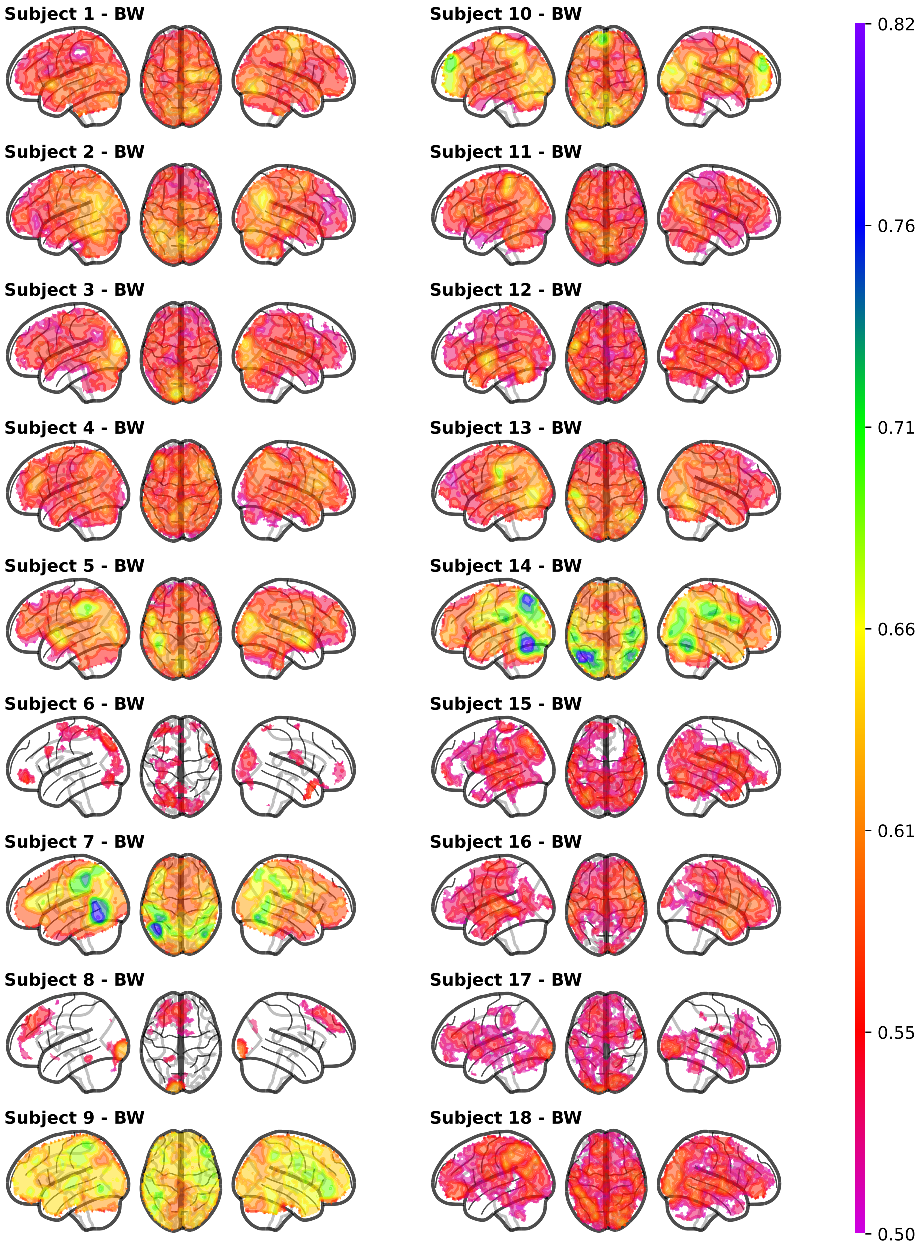


**Fig. C4**: Subject variability of the DA-enhanced searchlight accuracy with the BW technique. For each subject, the colour of each voxel represents the average accuracy over the 100 repetitions of the experiment in the regions where such accuracy is statistically significantly above the voxel-wise empirical null distribution.

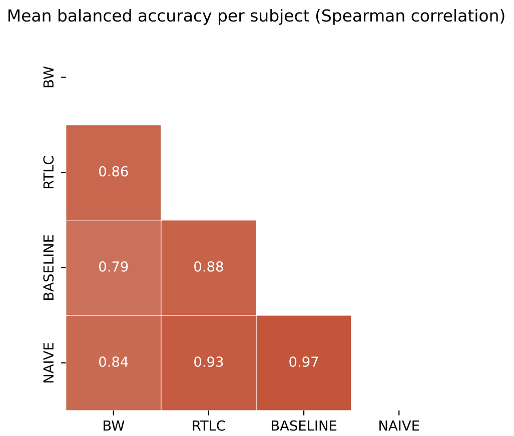


**Fig. C5**: Correlation between the searchlight results for individual subjects obtained in each searchlight procedure. Each method was represented by the average balanced accuracies for each subject.

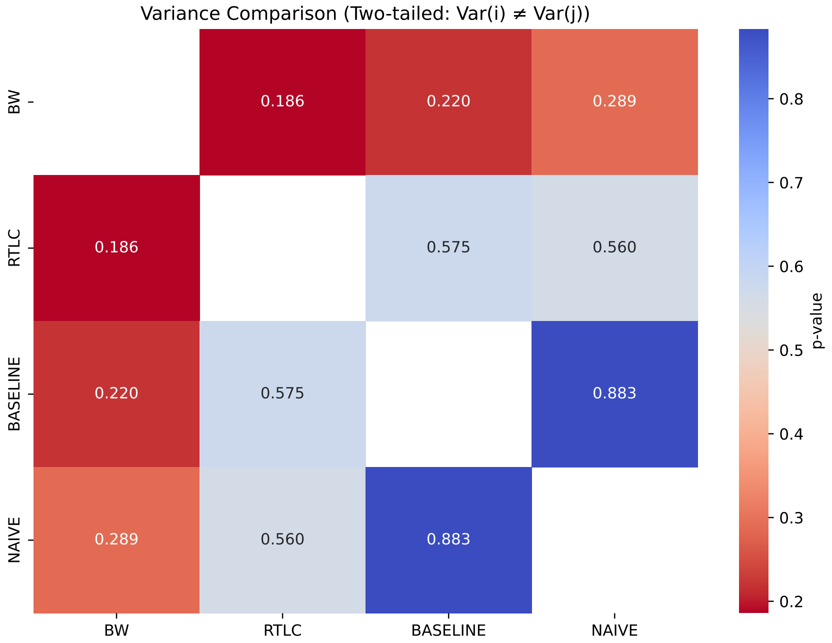


**Fig. C6**: Pairwise F-tests comparing the variance of the searchlight results accross subjects for the different searchlight procedures. Each method was represented by the average balanced accuracies for each subject, and the variance of each method was compared against the rest. p-values above 0.05 indicate a failure to reject the null hypothesis, which stated that there is a statistically significant difference in the variances.

## Appendix C.2 Results with the Balanced Weighting DA technique

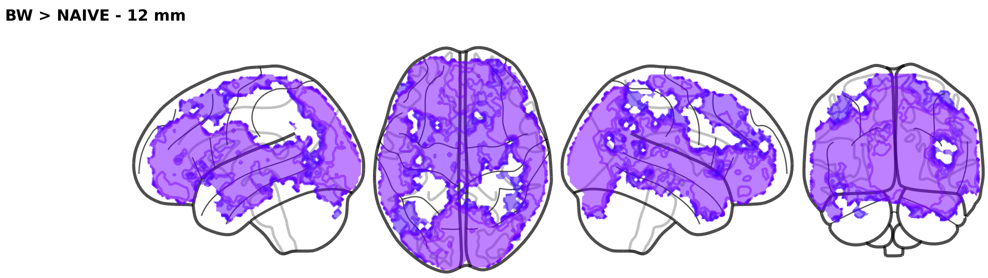


**Fig. C7**: Brain regions where BW decoding is significantly better than the naïve approach ($p < 0.05$, whole-brain corrected).

## Appendix C.3 Sensitivity analysis for the radius of the searchlight spheres

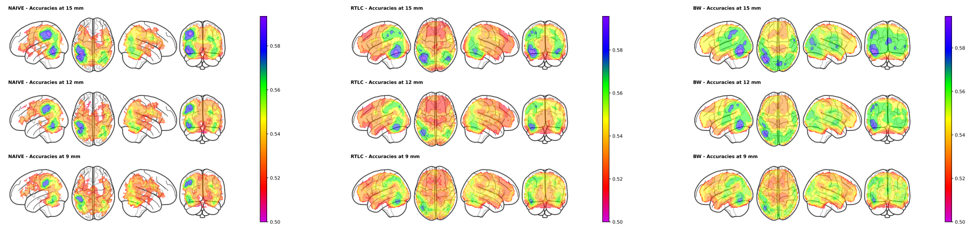

(a) NAIVE (b) RTLC (c) BW

**Fig. C8**: Balanced accuracies of the NAIVE and DA-enhanced searchlights for different sizes of the searchlight spheres, highlighting only the voxels where decoding is significantly better than the voxel-wise empirical null distribution ($p < 0.05$, whole-brain corrected).

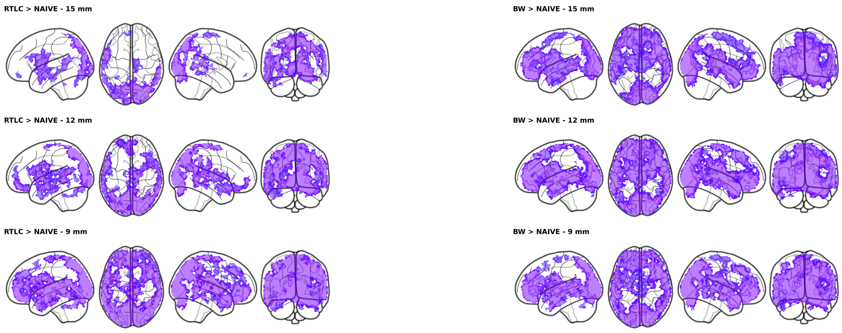


(a) RTLC (b) BW

**Fig. C9**: Brain regions where each DA-enhanced searchlight decoding is significantly better than the NAIVE approach ($p < 0.05$, whole-brain corrected), for different sizes of the searchlight spheres.
